# Association of virome dynamics with mosquito species and environmental factors

**DOI:** 10.1186/s40168-023-01556-4

**Published:** 2023-05-08

**Authors:** Qing Liu, Feng Cui, Xiang Liu, Yumei Fu, Wenjing Fang, Xun Kang, Hong Lu, Siping Li, Biao Liu, Wei Guo, Qianfeng Xia, Le Kang, Feng Jiang

**Affiliations:** 1 Beijing Institutes of Life Science, Chinese Academy of Sciences, Beijing, China; 2 State Key Laboratory of Integrated Management of Pest Insects and Rodents, Institute of Zoology, Chinese Academy of Sciences, Beijing, China; 3 CAS Center for Excellence in Biotic Interactions, University of Chinese Academy of Sciences, Beijing, China; 4 NHC Key Laboratory of Tropical Disease Control, Key Laboratory of Tropical Translational Medicine of Ministry of Education, School of Tropical Medicine, Hainan Medical University, Haikou, Hainan China

**Keywords:** Mosquito, Virome, RNA virus, Food source, Environment

## Abstract

**Background:**

The pathogenic viruses transmitted by mosquitoes cause a variety of animal and human diseases and public health concerns. Virome surveillance is important for the discovery, and control of mosquito-borne pathogenic viruses, as well as early warning systems. Virome composition in mosquitoes is affected by mosquito species, food source, and geographic region. However, the complex associations of virome composition remain largely unknown.

**Results:**

Here, we profiled the high-depth RNA viromes of 15 species of field-caught adult mosquitoes, especially from *Culex*, *Aedes*, *Anopheles*, and *Armigeres* in Hainan Island from 2018 to 2020. We detected 57 known and 39 novel viruses belonging to 15 families. We established the associations of the RNA viruses with mosquito species and their foods, indicating the importance of feeding acquisition of RNA viruses in determining virome composition. A large fraction of RNA viruses were persistent in the same mosquito species across the 3 years and different locations, showing the species-specific stability of viromes in Hainan Island. In contrast, the virome compositions of single mosquito species in different geographic regions worldwide are visibly distinct. This is consistent with the differences in food sources of mosquitoes distributed broadly across continents.

**Conclusions:**

Thus, species-specific viromes in a relatively small area are limited by viral interspecific competition and food sources, whereas the viromes of mosquito species in large geographic regions may be governed by ecological interactions between mosquitoes and local environmental factors.

Video Abstract

**Supplementary Information:**

The online version contains supplementary material available at 10.1186/s40168-023-01556-4.

## Introduction

Mosquito-borne pathogens pose an immense threat to public health threat in nearly half the human population and many nonhuman vertebrates [1]. In fact, these pathogenic viruses represent only a minor fraction of the viromes of mosquitoes. Nonpathogenic viruses have a critical impact on vector competence and modulate the infection outcomes of pathogenic viruses, although they do not directly threaten public health [2]. Virome surveillance has considerably extended our understanding of the entire repertoire of mosquito-associated viruses, because of its capacity to monitor both known zoonotic disease pathogens and newly emerged RNA viruses [3]. Thus, exploring the virome in mosquitoes has important significance in reflecting viral origin, evolution, diversity, and distribution. With extensive investigation, new unclassified viruses in mosquitoes have been constantly detected in recent years. Thus, knowledge of mosquito viromes is far from being completely understood [4].

Many virome studies have demonstrated that the virome composition in mosquitoes is principally structured by host mosquito taxon in a relatively limited area [5–10]. A marked difference of the viromes between *Aedes aegypti* and *Culex* (*Cx*) *quinquefasciatus* was revealed in a shared region less than 15 km wide [10]. The viromes in *Armigeres subalbatus*, *Cx. fuscocephala*, and *Mansonia uniformis* are defined primarily by the host mosquito species rather than by geographical location at a 20-km scale [11]. However, the two *Culex* species, with overlapping geographical distributions within 600 km, share numerous viruses [8]. In fact, the role of the mosquito species barrier in shaping virome composition is restricted. The effects of the host species barrier on interspecies virome composition need to be taken into consideration within the scope of geography [8]. However, the geographic contributions in a large-scale region to virome changes have been largely overlooked. In most virome studies aimed at virus discovery, a large number of mosquito individuals from multiple locations or multiple species were combined into a few pooled samples. The scarcity of location and host-specificity information limits our understanding of virome composition in relation to the geographical distribution of their host mosquitoes. Therefore, the investigation of virome stability in single mosquito species in spatially hierarchical geographical ranges is needed.

Food is one of the important ways for mosquitoes to acquire RNA viruses in addition to their symbiotic viruses. Mosquitoes harbor pathogenic viruses, which can replicate and be transmitted by mosquito vectors to vertebrate hosts via the bloodmeal. They also carry plant viruses that are not capable of replicating in mosquitoes and may be present in plant liquids they feed upon [12, 13]. Since an increase in the feeding rate of mosquitoes promotes the chance of acquiring and spreading the RNA virus, food source preference is a critical factor affecting virome composition [14]. Therefore, establishing the associations between mosquito species and their food sources for RNA viruses facilitates understanding the path of RNA virus spread. However, the complicated associations in nature are highly unpredictable and remain largely unknown [15].

Virome dynamics are dependent on the interactions between mosquitoes and their environments. Except for vertically transmitted viruses, the main components of the virome in mosquitoes are transmitted horizontally in the environment [16]. Because local habitat and geography are variable in different natural environments, the comparison of viromes among different geographic regions can uncover environmental contributions to virome composition. Hainan Island, a tropical island that is located in the northern South China Sea, has a remarkable diversity of animals and plants [17]. Tropical islands are hotspots for the emergence of mosquito-borne viruses due to sylvatic spillover events resulting from increases in commerce, travel, and urbanization [18]. In fact, mosquito-borne infectious diseases, such as malaria, dengue fever, and filariasis, were historically prevalent in Hainan Island [17, 19]. Therefore, virome characterization and surveillance of mosquitoes in Hainan Island could provide important cues for the prevention of pathogen transmission and early warning programs for preexisting arboviruses.

We conducted large-scale mosquito collection and virome identification in 13 counties covering the main ranges of Hainan Island from 2018 to 2020. The viromes in 15 mosquito species across different locations and years were quantitatively analyzed. In particular, we explored the co-occurrent associations among mosquito species, food sources, and RNA viruses. Furthermore, we compared the virome composition and food source of the same species in different hierarchical geographic regions. Together, our results reveal that environmental factors contribute much more than mosquito species in shaping the viromes in mosquitoes.

## Results

### Data summary of mosquito species and RNA viruses

**Fig. 1:**
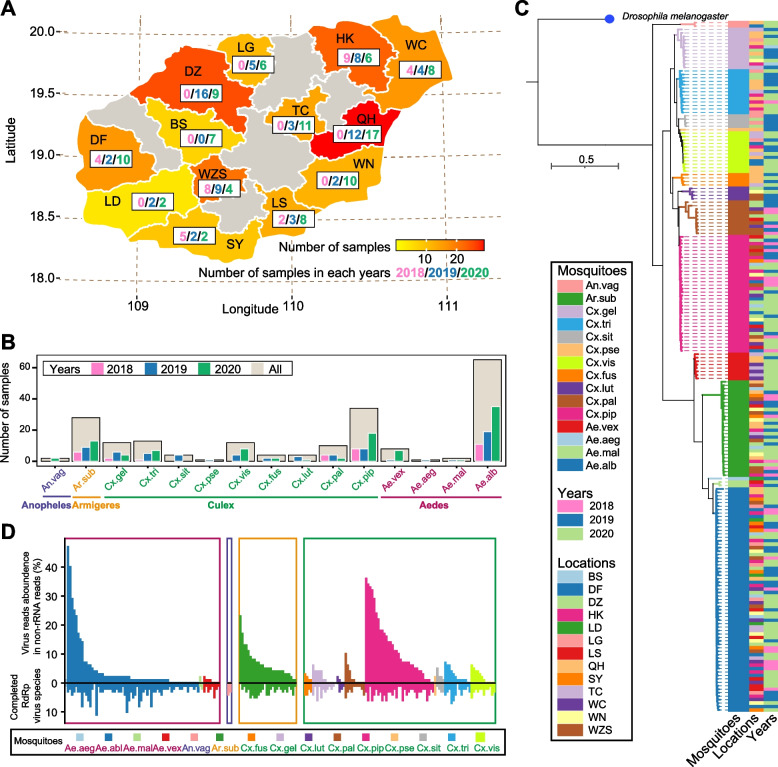
Mosquito sequencing and sample distribution. **A** Sampling locations of mosquito species from 2018 to 2020 in Hainan Island, China. **B** The number of mosquito samples collected across mosquito species from 2018 to 2020 in Hainan Island, China. **C** Phylogenetic tree based on nucleotide sequences of BUSCO single-copy orthologs using maximum likelihood inference. **D** Viral abundance (top) and the number of identified RNA viruses with complete sequences of RNA-dependent RNA polymerase (bottom). Ae.alb, *Aedes albopictus*; Ae.aeg, *Aedes aegypti*; Cx.pip, *Culex pipiens quinquefasciatus*; Ar.sub, *Armigeres subalbatus*; Cx.tri, *Culex tritaeniorhynchus*; Cx.pal, *Culex pallidothorax*; Ae.vex, *Aedes vexans*; Cx.vis, *Culex vishnui*; Cx.fus, *Culex fuscocephala*; Cx.sit, *Culex sitiens*; Cx.pse, *Culex pseudovishnui*; An.vag, *Anopheles vagus*; Cx.lut, *Culex (Lutzia) fuscanus*; Ae.mal, *Aedes malayensis*; Cx.gel, *Culex gelidus*. BS, Baisha; DF, Dongfang; DZ, Danzhou; HK, Haikou; LD, Ledong; LG, LinGao; LS, Lingshui; QH, Qionghai; SY, Sanya; TC, Tunchang; WC, Wenchang; WN, Wanning; WZS, Wuzhishan

Field-caught adult mosquitoes were collected in 13 counties in Hainan Island in July from 2018 to 2020 (Fig. 1A). The collected mosquitoes were subjected to species identification based on the combination of morphology and DNA barcoding. We included the mosquito species for which these two methods showed identical results for the subsequent sequencing procedures. A total of 15 mosquito species, including nine *Culex* (*Cx*), four *Aedes* (*Ae*), one *Anopheles* (*An*), and one *Armigeres* (*Ar*), were used for virome sequencing, which was carried out on 200 pools of mosquitoes (Fig. 1B), with each pool containing three individuals of each species per location. The species-level phylogeny was reconstructed using Benchmarking Universal Single-Copy Orthologs (BUSCO) to consolidate the species identification results. The hierarchical clustering pattern of mosquito genera and species obtained using BUSCO gene sets agreed with the species identification results obtained using the combined approach for all 200 sequencing libraries (Fig. 1C).

A total of 6.76 terabytes of virome sequencing reads (average of 225,352,063 reads per pool) were generated and assembled de novo into 120,834,188 contigs (average of 604,171 contigs per pool) for virus discovery and characterization (Supplementary Table 1). Because the presence of fragmented RNA viruses hampers the precise quantification of RNA viruses, we used the RNA-dependent RNA polymerase (RdRp) sequence to quantify the virus species. We found a high diversity of RNA viruses comprising 96 species based on an RdRp amino acid (aa) identity threshold of 80% in comparison to their closest known homologs (Supplementary Table 2). The number of RNA viruses in different mosquito species ranged from 1 to 26, with the highest number in *Ae. albopictus* and the lowest in *Ae. aegypti* (Supplementary Table 3). We failed to find any known pathogenic arboviruses related to human and vertebrate diseases in mosquitoes. The virus read abundance among non-rRNA reads was highly variable across mosquito species (Fig. 1D). The number of RNA viruses detected was significantly linearly correlated with virus read abundance (Pearson correlation test, *P* < 0.05), emphasizing the capacity for virus detection based on the high-depth sequencing data in this study.

### Phylogeny and genome annotation for novel RNA viruses

We generated phylogenetic trees using the protein sequences of RdRp to confirm the taxonomic phylogeny of the 57 known and 39 potentially novel RNA viruses (Figs. 2A–2F; see Supplementary Figures 1–19 for detailed phylogenies). The 39 novel RNA viruses belonging to five phyla (*Lenarviricota*, *Pisuviricota*, *Kitrinoviricota*, *Duplornaviricota*, and *Negarnaviricota*) comprise putative members of five unclassified viruses and 15 different viral families (Fig. 2G): *Rhabdoviridae*, *Phenuiviridae*, *Orthomyxoviridae*, *Phasmaviridae*, *Xinmoviridae*, *Totiviridae*, *Sedoreoviridae*, *Spinareoviridae*, *Nodaviridae*, *Endornaviridae*, *Partitiviridae*, *Dicistroviridae*, *Iflaviridae*, *Polycipiviridae*, and *Narnaviridae*.

**Fig. 2:**
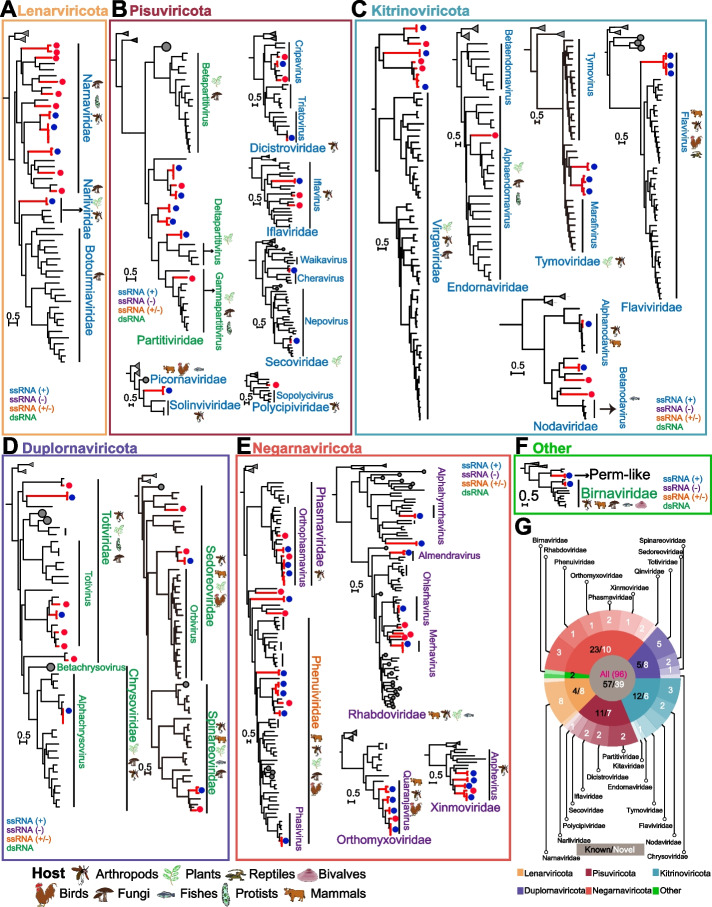
Maximum likelihood phylogenies of RNA viruses identified in this study based on protein sequences of full-length RNA-dependent RNA polymerase in the phyla *Lenarviricota* (**A**), *Pisuviricota* (**B**), *Kitrinoviricota* (**C**), *Duplornaviricota* (**D**), *Negarnaviricota* (**E**), and others (**F**). Branch lengths are indicated by the scale bar. The RNA viruses identified in this study are labeled by red branches. The red circles represent viruses newly identified in this study, and the blue circles represent previously published viruses. **G** Taxonomic distribution of RNA viruses discovered in this study

#### *Lenarviricota*

**Fig. 3:**
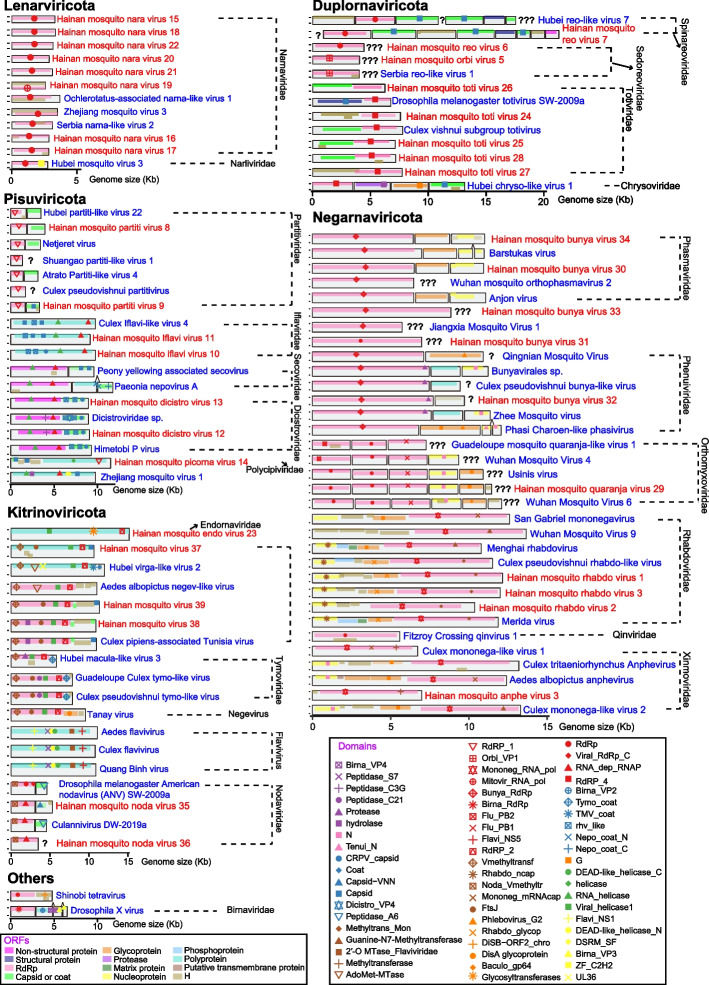
Genome structure of RNA viruses in the phyla *Lenarviricota*, *Pisuviricota*, *Kitrinoviricota*, *Duplornaviricota*, *Negarnaviricota*, and others. The red and blue colors represent the nomenclature of novel RNA viruses identified in this study and the previously reported RNA viruses, respectively. The symbols “?” and “???” represent one and multiple unknown segment(s) in the genomes of segmented RNA viruses, respectively. Two fragments anchored to the previously reported RNA viruses are labeled by the symbol “^”

Eight novel viruses and three known viruses were identified in the family *Narnaviridae* (Fig. 2A), whereas one known virus in the family *Narliviridae* was recently classified as a new family (Supplementary Figure 1) [20]. These two families are members of the phylum *Lenarviricota*, which is the basal branch of RNA viruses representing a major evolutionary change from prokaryotic to eukaryotic RNA viruses [20, 21]. The family *Narnaviridae* has been reported to use fungi, protists, diatoms, and invertebrates as hosts [20, 22–25]. Unlike other *Narnaviridae* viruses, the four RNA viruses, Ochlerotatus-associated narna-like virus 1, Zhejiang mosquito virus 3, Hainan mosquito nara virus 19, and Serbia narna-like virus 2, have not only one RdRp ORF in the sense strand but also a long, uninterrupted ORF in the reverse complement strand (Fig. 3). In addition, the RdRp ORF containing the Mitovir_RNA_pol domain is encoded by the standard genetic code rather than mitochondrial genetic code in Hainan mosquito nara virus 19. These two genomic signatures, ORF in the reverse complement strand and Mitovir_RNA_pol domain, may be originally from the family *Mitoviridae* [20, 25].

#### *Pisuviricota*

Five known viruses and two new viruses discovered here are related to the bi-segmented dsRNA viruses of the family *Partitiviridae* (Fig. 2B and Supplementary Figure 2). Except for Culex pseudovishnui partitivirus and Hainan mosquito para virus 9, the remaining five viruses showed <30% amino acid (aa) identities of RdRp with their closest known viruses, indicating that these five viruses are from putative unclassified lineages (Supplementary Table 2). Ten viruses (five known viruses and five novel viruses) were reported in the diverse clusters belonging to various families among the ssRNA+ viruses of order *Picornavirales*: *Iflaviridae*, *Secoviridae*, *Dicistroviridae*, and *Polycipiviridae* (Supplementary Figures 3–6) [21, 22]. In contrast to the family *Secoviridae* infecting plants, the remaining three viral families can infect only arthropod species [21, 22]. The Peptidase_C3G domain could not be identified in the Hainan mosquito para virus 13 and Himetobi P virus from the family *Dicistroviridae* (Fig. 3) [26]. Compared to Culex Iflavi-like virus 4, Hainan mosquito para virus 11 and Hainan mosquito para virus 10 show multiple capsid-type domain rearrangements. Zhejiang mosquito virus 1 was first reported as a Kelp Fly virus-related clade [25]. Compared to the closest homologs in the family *Solinviviridae*, this virus species shows less than 20% aa identity to RdRp and has a different genome architecture, suggesting that the Kelp Fly virus related clade is a new virus family.

#### *Kitrinoviricota*

A total of 18 viruses identified here have a close phylogenetic relationship to the *Kitrinoviricota* phylum (Fig. 2C, Fig. 3, and Supplementary Figures 8–12), in which most members have positive-strand RNA virus genomes [ssRNA(+)]. Six of them, showing highly divergent from known viruses, could not be grouped with the current virus classification scheme (Supplementary Table 2). A conserved RNA replication module including capping enzyme, superfamily 1 helicase, and RdRp is present in viruses belonging to the class *Alsuviricetes* except for *Endornaviridae* [21, 25]. Although the newly discovered virus Hainan mosquito endovirus 23 shared an only 20.6% aa identity of RdRps with the virus Phaseolus vulgaris alphaendornavirus 1, both virus species have a full-length polyprotein encoding viral helicase1, glycosyltransferases, and RdRP_2 domains (Fig. 3). Three previously characterized viruses (Hubei virga-like virus 2, Aedes albopictus negev-like virus, and Culex pipiens-associated Tunisia virus) and three new viruses (Hainan mosquito virus 37, Hainan mosquito virus 38, and Hainan mosquito virus 39) were monophyletic and distantly related to the family *Virgaviridae* (Supplementary Figure 9). Differing from Cucumber green mottle mosaic virus and Tobacco rattle virus in the family *Virgaviridae*, these six viruses have a unique FtsJ family methyltransferase domain, suggesting that they form a potential new virus family [25]. Two new viruses, Hainan mosquito noda virus 35 and Hainan mosquito noda virus 36, float between the *Alphanodavirus* and *Betanodavirus* genera in the family *Nodavirida*e (Supplementary Figure 12). They are phylogenetically close to Nelson wasp-associated virus 3 and Bat guano associated nodavirus GF-4n, respectively. These results reinforce the previous proposal that there should be more than two genera in the family *Nodavirida*e [27].

#### *Duplornaviricota*

Except for those in the phylum *Pisuviricota*, all double-stranded RNA viruses are present in the phylum *Duplornaviricota*. Five novel viruses and two known viruses in this study were from the family *Totiviridae*, which is a family of the class *Chrymotiviricetes* under the phylum *Duplornaviricota* (Fig. 2D, Fig. 3, and Supplementary Figure 13). Three novel viruses, including Hainan mosquito toti virus 25, Hainan mosquito toti virus 28, and Hainan mosquito toti virus 24, have closer phylogenetic relations with other toti-like viruses reported to use mosquitoes as hosts [9, 25, 28, 29]. Additionally, these viruses have a longer branch length than Ustilago maydis virus H which uses fungi as hosts, suggesting that they are mosquito-specific viruses. In the order *Reovirales*, the low aa identities with their closest homologs in the three viruses, Hainan mosquito reo virus 6 (30.1%), Hainan mosquito orbi virus 5 (59.9%), and Hainan mosquito reo virus 7 (65.0%), suggest that they are novel viruses at the species rank (Supplementary Table 2). Although only one genome segment harboring the full-length RdRp protein sequence was detected, we found the Orbi_VP1 domain in the two viruses (Fig. 3), named Serbia reo-like virus 1 and Hainan mosquito orbi virus 5, both of which belong to the genus *Orbivirus* within the family *Sedoreoviridae* (Supplementary Figure 14).

#### *Negarnaviricota*

All five newly discovered viruses and nine known viruses belong to the order *Bunyavirales* (Figs. 2E and 3). A novel virus, Hainan mosquito bunya virus 33 and Beihai sesarmid crab virus 5, formed a monophyletic branch between *Phasmaviridae* and *Leishbuviridae*, suggesting a new virus family (Supplementary Figure 15) [25]. We also identified at least six genome segments for a newly discovered virus Hainan mosquito quaranja virus 29, which belongs to the genus *Quaranjavirus* in the family *Orthomyxoviridae* (Supplementary Figure 16). Several viruses in this genus have been reported to be associated with mass avian die-offs and unexplained febrile illness in children [30, 31]. We identified three novel and five known negative-strand mononegaviruses belonging to *Rhabdoviridae* (Supplementary Figure 17), a virus family that infects mammals, arthropods, plants, or fishes [32]. The known virus Culex pseudovishnui rhabdo-like virus isolated from *Culex vishnui* shows high RdRp aa identity and genome organization similarity with the North Creek virus (Fig. 3 and Supplementary Table 2). Additionally, the novel viruses Hainan mosquito rhabdo virus 1, Hainan mosquito rhabdo virus 2, and Hainan mosquito rhabdo virus 3 are phylogenetically close to *Ohlsrhavirus* and *Merhavirus* (Supplementary Figure 17). North Creek virus and Riverside virus 1 in the genus *Ohlsrhavirus*, and Culex tritaeniorhynchus rhabdovirus in the genus *Merhavirus*, can infect mammalian cell lines in vitro [33], suggesting that the pathogenic potential of these four viruses deserves further investigation.

### Associations of mosquito RNA viruses with food sources

**Fig. 4:**
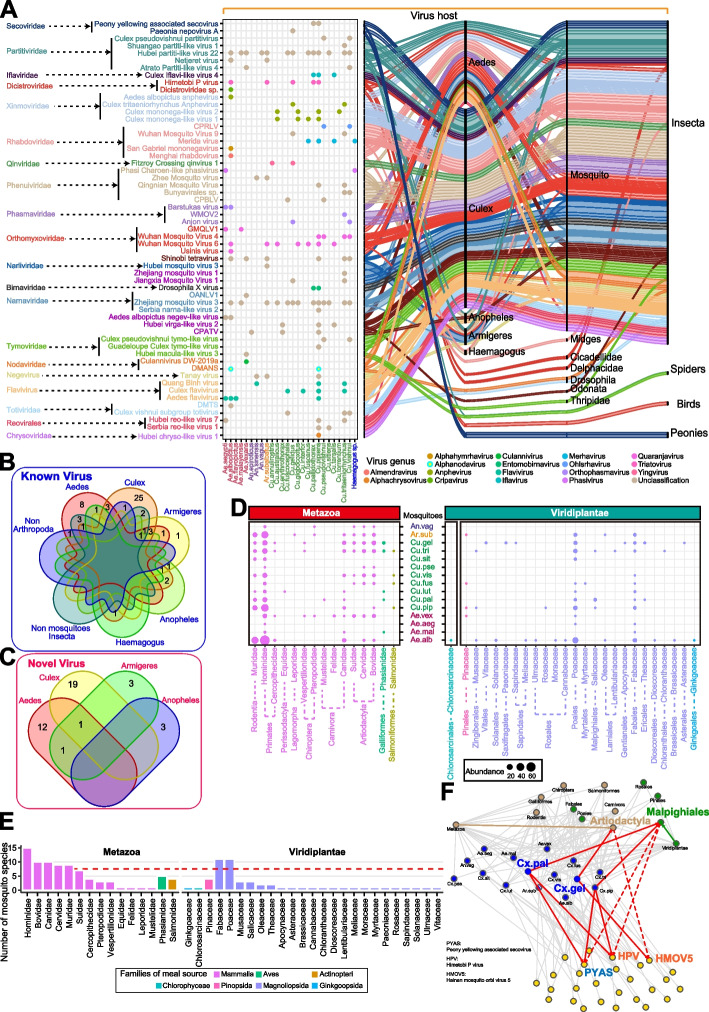
Associations among RNA virus, mosquito species, and food sources. **A** A tripartite network to visualize the associations among the known viruses identified in this study, mosquito species, and their potential animal and plant hosts using a combination of our mosquito data and the host‒virus records in the NCBI database. The host records in the NCBI database were determined where the species were subjected to virus assay. **B** Venn diagram describing the RNA viruses shared among animal and plant hosts for the known viruses. **C** Venn diagram describing the RNA viruses shared among the novel viruses. **D** The bubble plot shows the distribution of food sources derived from animals (left) and plants (right) across mosquito species. **E** Classification of food sources derived from animals (top) and plants (bottom) in mosquitoes. **F** Network geometry of associations among RNA viruses, mosquito species, and food sources. The red dotted line indicates the significant co-occurrence association of the RNA viruses and the food sources identified in this study. Statistical significance was determined by a hypergeometric test (*P* < 0.01). Ae.alb, *Aedes albopictus*; Ae.aeg, *Aedes aegypti*; Cx.pip, *Culex pipiens quinquefasciatus*; Ar.sub, *Armigeres subalbatus*; Cx.tri, *Culex tritaeniorhynchus*; Cx.pal, *Culex pallidothorax*; Ae.vex, *Aedes vexans*; Cx.vis, *Culex vishnui*; Cx.fus, *Culex fuscocephala*; Cx.sit, *Culex sitiens*; Cx.pse, *Culex pseudovishnui*; An.vag, *Anopheles vagus*; Cx.lut, *Culex (Lutzia) fuscanus*; Ae.mal, *Aedes malayensis*; Cx.gel, *Culex gelidus*. CPRLV, Culex pseudovishnui rhabdo-like virus; CPBLV, Culex pseudovishnui bunya-like virus; WMOV2, Wuhan mosquito orthophasmavirus 2; GMQLV1, Guadeloupe mosquito quaranja-like virus 1; OANLV1, Ochlerotatus-associated narna-like virus 1; CPATA, Culex pipiens-associated Tunisia virus; DMANS, Drosophila melanogaster American nodavirus (ANV) SW-2009a; DMTS, Drosophila melanogaster totivirus SW-2009a

Based on a combination of our data and the virus records from the NCBI database, we constructed a tripartite network to visualize the associations of the known viruses with mosquito species and their potential non-mosquito hosts derived from animals and plants (Fig. 4A). As expected, a large majority of the known viruses were only exclusively associated with mosquitoes at the genus level, suggesting host specificity of these viruses (Fig. 4B). The host specificity was also observed for the novel viruses (Fig. 4C). Several of these viruses were associated with multiple species of insects from Diptera, Hemiptera, Odonata, and Thripidae, suggesting cross-species horizontal transmission between insect hosts. For example, Himetobi P virus was detected in both *Aedes* and several planthopper species [34]. Unexpectedly, we found that two viruses (Dicistroviridae sp. and Peony yellowing associated secovirus), which were detected only in bird vents (GenBank: QJI52079.1) and peonies (GenBank: QNN26213.1, Paeoniaceae, Viridiplantae), were found the first time found in the mosquitoes *Ae. albopictus* and *Cx. pallidothorax*, respectively. These results implied a possible route of spread for RNA viruses between mosquitoes and animals/plants.

Because identifying the linkage of mosquito species and food sources can provide the information regarding the reservoir of RNA viruses and transmission paths, we explored the possibility of determining the food sources using virome data in mosquitoes. The assignment of the non-mosquito contigs to the lowest common ancestor (LCA) to the kingdom Metazoa showed that the bloodmeal hosts belonged to three classes, Mammalia, Aves, and Actinopteri (Fig. 4D). At the family level, the mammalian families, including Hominidae, Bovidae, and Canidae, represented the top three sources of bloodmeals (Fig. 4E). Thus, the mosquito species in Hainan Island commonly feed on humans and secondarily on *Bos* species, likely attributed to the availability of food sources. In particular, host food sources from Salmonidae, a family of ray-finned fishes, were detected only in *Culex* species, including *Cx. tritaeniorhynchus*, *Cx. vishnui*, *Cx. fuscocephala*, and *Cx. pipiens quinquefasciatus*. Therefore, mosquito species showed considerable preferences for bloodmeals from ectothermic hosts.

Because of the existence of plant viruses in mosquitoes, we determined if the mosquitoes could use the plant sources by assigning non-mosquito contigs to the LCA to the kingdom Viridiplantae. In addition to a few cases in the families Ginkgoopsida and Pinopsida of a limited number of mosquito species, we found that flowering plants (Magnoliopsida) were the major plant-derived diet component of mosquitoes (Fig. 4D). Although all the samples were collected from mosquito adults, we also detected green algae (the families Chlorosarcinaceae and Uronemataceae), which are considered nutritious foods for mosquito larvae in aquatic environments [35]. The most frequently observed plant families were the Fabaceae and Poaceae, which mainly include *Lupinus*, *Glycine*, and *Oryza* species (Fig. 4E). The saturation analysis revealed that the detected family number increased with increasing library numbers, emphasizing the power of high-depth sequencing data for food source detection (Supplementary Figure 20). Because index hopping results in the misassignment of reads among different libraries during demultiplexing, we randomly validated the plant-derived RNAs in the same samples that were subjected to virome sequencing using the PCR Sanger sequencing method (Supplementary Figure 21). In addition, *Keteleeria hainanensis* (Pinaceae), an endemic plant species in Hainan Island, was detected in the assemblies and was further verified using PCR Sanger sequencing (Supplementary Figure 21). Thus, the results excluded the possibility that the plant-derived RNAs in mosquito virome sequencing data were artifacts associated with sequencing technology.

To assess the significant associations between RNA viruses and their food sources, we performed hypergeometric tests between each family of food sources and each RNA virus across all the mosquito sequencing libraries (Fig. 4F). We statistically confirmed three significant co-occurrent associations of specific RNA viruses with their food sources. Hainan mosquito orbi virus 5, a novel orbivirus identified in this study, was detected in *Cx. gelidus*. Most likely, the *Cx. gelidus* mosquitoes took bloodmeals from Bovidae species. In *Cx. pallidothorax* and *Cx. pipiens quinquefasciatus*, we found two plant viruses, Peony yellowing associated secovirus and Himetobi P virus, which were significantly co-occurred with the Salicaceae species, the willow family of flowering plants. Peony yellowing-associated secovirus and Himetobi P virus were previously detected only in peony (Paeoniaceae) plants and planthopper species (Hemiptera), respectively [36]. Our results extended the host range of these two RNA viruses. Thus, these results showed that the food sources can be identified via virome sequencing to establish associations with food sources and mosquito species for RNA viruses.

### Virome variation of dominant mosquito species in Hainan Island

**Fig. 5:**
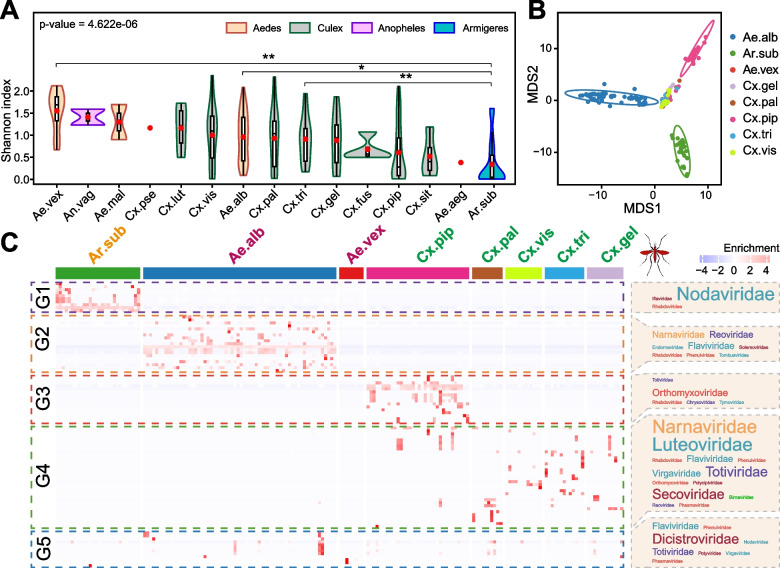
Virome composition across mosquito species. **A** Shannon index of virus operational taxonomic units (OTUs) across mosquito species. Statistically significant differences were detected using a Kruskal‒Wallis test in cases of multiple comparisons. In pairwise comparisons, significant differences were detected by pairwise Wilcoxon rank sum tests. *P < 0.05; **P < 0.001. **B** Multiple scaling analysis for virome composition across mosquito species using a Euclidean distance matrix. **C** Heatmap plot showing the enrichment of normalized expression of virus OTUs across different samples of mosquito species. The virus OTUs were clustered using K-means based on Euclidean distances. The font size of the virus family in the right text box was scaled to the number of identified RNA viruses in each virus family. Ae.alb, *Aedes albopictus*; Ae.aeg, *Aedes aegypti*; Cx.pip, *Culex pipiens quinquefasciatus*; Ar.sub, *Armigeres subalbatus*; Cx.tri, *Culex tritaeniorhynchus*; Cx.pal, *Culex pallidothorax*; Ae.vex, *Aedes vexans*; Cx.vis, *Culex vishnui*; Cx.fus, *Culex fuscocephala*; Cx.sit, *Culex sitiens*; Cx.pse, *Culex pseudovishnui*; An.vag, *Anopheles vagus*; Cx.lut, *Culex (Lutzia) fuscanus*; Ae.mal, *Aedes malayensis*; Cx.gel, *Culex gelidus*

*Ae. albopictus*, *Cx. pipiens quinquefasciatus*, and *Ar. subalbatus* were the most prevalent mosquito species in Hainan Island [17, 37]. We compared virome diversity (Shannon index) across mosquito species in Hainan Island. The alpha diversities of virus operational taxonomic units (OTUs) varied among the mosquito species, with the highest diversity observed in *Ae. vexans* and the lowest in *Ar. subalbatus* (Fig. 5A). Because alpha diversity is reflected by the observed richness and abundance distribution of viruses, we separately determined the observed richness and abundance of RNA viruses across mosquito species. Compared to other mosquito species, *Ar. subalbatus* showed a higher abundance of a few virus species (Supplementary Figure 22), indicating an uneven abundance distribution of its virus OTUs.

To examine the effects of mosquito species and location on virome composition, multidimensional scaling was performed to determine their correlation. We found that the virome composition of the three species of mosquitoes, *Ae. albopictus*, *Cx. pipiens quinquefasciatus*, and *Ar. subalbatus*, was largely structured by the mosquito species rather than the location (Fig. 5B and Supplementary Figure 23). In the three mosquito species, we identified a total of 54 RNA viruses, of which 46 (85.19%) were species-specific viruses (Supplementary Figure 24). Furthermore, the K-means clustering of virus OTU abundances revealed five virus clusters that were associated with distinct virus communities (Fig. 5C). Three virus clusters were predominantly associated with *Ae. albopictus*, *Cx. pipiens quinquefasciatus*, and *Ar. subalbatus.* The *Flaviviridae*, *Orthomyxoviridae*, and *Nodaviridae* families were the most abundant in the corresponding mosquito species: *Ae. albopictus*, *Cx. pipiens quinquefasciatus*, and *Ar. Subalbatus*, respectively. The alpha diversity (Supplementary Figure 25, Kruskal‒Wallis test, *Ps* > 0.05) of the virome did not differ across the locations in Hainan Island.

We found a large degree of viruses sharing within mosquito species across the different locations (Fig. 5C) and different years (Supplementary Figure 24), indicating the existence of a species barrier in virome composition in Hainan Island as an independent geographic region. Therefore, the species-specific viromes were attributed to their unique virus representatives in *Ae. albopictus*, *Cx. pipiens quinquefasciatus*, and *Ar. subalbatus*. Species-specific persistence was observed in a few virus species across the years, suggesting that these viruses are maintained by vertical transmission. These vertically transmitted viruses include (1) Guapiacu virus, Guangzhou sobemo-like virus, Aedes albopictus negev-like virus, Hainan mosquito reo virus 6, Wenzhou sobemo-like virus 4, Usinis virus, and Riboviria sp. in *Ae. albopictus*; (2) Wuhan Mosquito Virus 6 and Hubei partiti-like virus 22 in *Cx. pipiens quinquefasciatus*; and (3) Hubei partiti−like virus 22, Hubei sobemo−like virus 41, Hainan mosquito virus 1, and Riboviria sp. in *Ar. subalbatus*. The mosaic distribution pattern for a large portion of viruses within species-specific viromes implies that these viruses were derived from the specific interactions between mosquito feeding and environments (Fig. 5C). Thus, the species-specific viromes in Hainan Island are likely to be composed of vertically transmitted and environment-derived RNA viruses.

### Virome variation in hierarchical geographical regions

Because species-specific viromes exist in Hainan Island, we attempted to determine whether the virome composition of a mosquito species varied as it gradually expanded the geographic range. We pooled all libraries for a mosquito species into a single dataset to compare the virome composition between different geographic regions. Only *Ae. albopictus* (USA) and *Cx. pipiens quinquefasciatus* (Yunnan of China and Sweden) were analyzed (Supplementary Table 4) based on the data availability of individual mosquito species and individual number information in public databases [8, 9]. To exclude the possibility of bias resulting from the differential involvement of sequencing depth and mosquito individuals, the virome data were randomly sampled to achieve equivalent data.

**Fig. 6:**
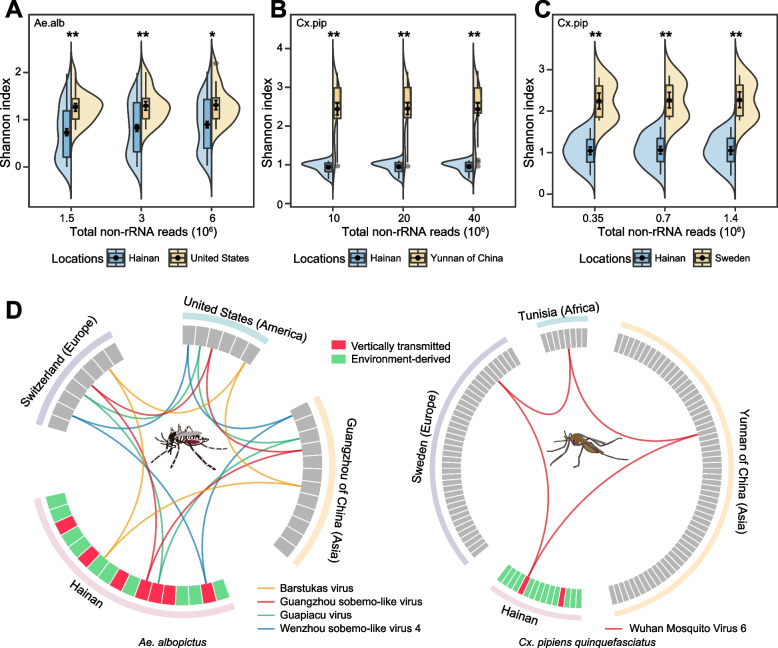
Virome composition of different continents. **A** Shannon index of virus OTUs of Hainan and California (USA, North America) in *Aedes albopictus*. **B** Shannon index of virus OTUs of Hainan and Yunnan (China, Asia) in *Culex pipiens quinquefasciatus*. **C** Shannon index of virus OTUs of Hainan and Sweden (Europe) in *Culex pipiens quinquefasciatus*. Stars indicate statistically significant differences between groups determined by pairwise Wilcoxon rank sum tests. The downsampling subset frequency was set to 25%, 50%, and 100% based on sequencing depth and the number of mosquito individuals involved. **D** Spatial associations of RNA viruses that are shared between different continents. Only the RNA viruses that were simultaneously detected on different continents are shown. Ae.alb, *Aedes albopictus*; Cx.pip, *Culex pipiens quinquefasciatus*

In *Ae. albopictus* and *Cx. pipiens quinquefasciatus*, we found that the alpha diversity in Hainan Island was significantly lower than that in the other three regions (Fig. 6A–C, Wilcoxon rank sum tests, *Ps* < 0.05). The results were also robust at 50% and 25% random downsampling rates. The lower diversity of viromes in Hainan Island resulted from the lower observed richness and abundance of viruses compared to those in the regions of America and Europe (Supplementary Figure 26). However, the difference in virus abundance between Hainan and Yunnan of China in *Cx. pipiens quinquefasciatus* was not significant. Thus, these results suggested the virome of tropical islands is less diverse than that of continental regions.

We further analyzed the presence of each virus OTU in hierarchical geographical regions worldwide for mosquito viromes sharing among different continental regions. The virome data of *Ae. albopictus* (Guangzhou of China, USA, and Switzerland) and *Cx. pipiens quinquefasciatus* (Yunnan of China, Tunisia, and Sweden) in three continental regions were analyzed (Supplementary Table 4). In *Ae. albopictus* and *Cx. pipiens quinquefasciatus*, only 23.53% (4 in 17) and 6.67% (1 in 15) of the viruses detected in Hainan Island were intersected with the viruses detected in other three continental regions, respectively. For *Ae. albopictus*, only one (Barstukas virus) of ten environment-derived viruses in Hainan Island was detected in the mosquito species of the other three continental regions. Three (Guapiacu virus, Guangzhou sobemo-like virus, and Wenzhou sobemo-like virus 4) of the seven vertically transmitted viruses in Hainan Island were shared by the *Ae. albopictus* mosquito from the other three continental regions*.* For *Cx. pipiens quinquefasciatus*, we did not detect any intersection of the 13 environment-derived viruses between Hainan Island and the other three continental regions (Fig. 6D and Supplementary Figure 24). Only one (Wuhan Mosquito Virus 6) of the two vertically transmitted viruses in Hainan Island was present in the *Cx. pipiens quinquefasciatus* mosquito from the other three continental regions*.* These results showed that very few environment-derived viruses are shared by the same mosquito species in different geographic regions. Therefore, species-specific viromes were not persistent in large geographic regions.

Because the acquisition of RNA viruses in mosquitoes is largely from foods in local environments, we compared the food source differences in single mosquito species in Hainan Island (Supplementary Figure 27). As expected, only 3 (20%; Fabaceae, Poaceae*,* and Musaceae) of 15 families in Viridiplantae were shared between the two mosquito species. We detected a total of 7 Metazoan families, but only 4 (Hominidae, Bovidae, Muridae, and Canidae) of them were shared between *Ae. albopictus* and *Cx. pipiens quinquefasciatus*. These results showed a considerable difference in the food sources of mosquito species in Hainan Island. We further expanded this analysis to mosquito species in different geographic regions (Supplementary Figure 28). In *Ae. albopictus* and *Cx. pipiens quinquefasciatus*, only one (Hominidae) of 11 Metazoan families and one (Muridae) of 14 Metazoan families were shared by the four continental regions studied. We did not detect any intersection of the families of Viridiplantae in the two mosquito species from the four continental regions, suggesting that the food source derived from green plants was more variable than that from animals.

## Discussion

We detected 57 known viruses and 39 novel viruses by exploring mosquito viromes in Hainan Island. The co-occurrent associations of RNA viruses with mosquito species and food sources suggested an environmental contribution to the acquisition and spread of viruses in mosquitoes. A large fraction of the viromes were persistent in the same mosquito species across different locations and years, indicating the species-specific stability of virome composition in Hainan Island. However, large-scale regional comparisons of the same mosquito species revealed entirely different virome compositions and food sources. Thus, our study suggested that the acquisition and spread of RNA viruses in ecosystems are dependent on endemic environmental associations and mosquito species barriers.

### Relevance of mosquito-borne viruses for human and animal health

In this study, we identified a large number of known and unknown viruses from 15 mosquito species in Hainan Island. We detected many viruses belonging to families of pathogenic arboviruses, such as *Flaviviridae*, *Sedoreoviridae*, *Orthomyxoviridae*, and *Rhabdoviridae*. Thus, these mosquitoes harbor a number of RNA viruses in Hainan Island, including viruses related to human and animal health. Members of these viral families were previously identified in other mosquito species and vertebrate hosts elsewhere, demonstrating that the host permissiveness of these virus families reflects their capacity to undergo interspecies transmission. In our study, the genus *Orbivirus* as a member of the family *Sedoreoviridae*, includes two known viruses, Serbia reo-like virus 1 and the novel virus Hainan mosquito orbi virus 5, both of which deserve high attention. In the genus *Orbivirus*, several infectious viruses such as the Peruvian horse sickness virus, Epizootic hemorrhagic disease virus, and bluetongue virus, can infect mammals, including cattle, deer, and sheep [38, 39]. Furthermore, Yunnan orbivirus has been detected in acute-phase serum samples obtained from hospitalized patients with fever and encephalitis [38]. These pathogenic viruses cluster with Serbia reo-like virus 1 and Hainan mosquito orbi virus 5, representing the basal lineage, to form a monophyletic group separated from the noninfectious virus lineage. Thus, further investigations aiming to decipher the epidemiology and clinical outcomes of these two RNA viruses in humans and livestock are necessary.

*Ae. aegypti* and *Ae. albopictus* are important vectors of dengue fever [40]. We did not detect the dengue virus in the two mosquito species, although 264 dengue fever cases were reported, and the outbreak lasted for over one month in 2019 in Hainan Island [19]. A similar result was reported previously, in which pathogenic arboviruses were not detected in field-caught mosquitoes using large-scale virome sequencing [9]. Mosquitoes harboring nonpathogenic viruses have low vectorial capacities, resulting in a reduction in the circulation and replication of arboviruses [41]. Low vectorial capacities of the mosquitoes harboring nonpathogenic viruses are attributed to superinfection exclusion and upregulation of antiviral immune responses of mosquitoes [42]. This implies that, despite a high tolerance for arbovirus infection, mosquitoes do not act as a long-term reservoir of mosquito-transmitted viruses.

### Spread paths of RNA viruses via food sources

Since several virus lineages infecting vertebrates and plants evolved from insect-specific viruses through host shifts [43], the association of mosquito species with RNA viruses and food sources paves the way to reconstruct the spread paths among them. Adult females of many hematophagous mosquito species are opportunistic blood feeders, while others show a feeding preference for specific animal food sources such as humans, bovines, birds, and amphibians [44]. Plant feeding in mosquitoes, most likely on floral nectar, honeydew, and fruit juices, also represents an important way to acquire sugars and nutrients [15]. In our study, food source analysis revealed that bloodmeals in mosquitoes are from a broad range of mammalian species, emphasizing a growing demand for the detection of RNA viruses in more mammals. Importantly, we detected one statistically significant co-occurrence between *Bovidae* species and a novel orbivirus, Hainan mosquito orbi virus 5. This was consistent with a previous report of orbiviruses, which can infect ruminants and result in bluetongue disease, African horse sickness, and epizootic hemorrhagic disease [45]. Thus, this result suggests the co-occurrence analysis using virome approaches is predictively valid in exploring the spread paths of viruses. In addition, the plant virus Peony yellowing associated secovirus co-occurred with the willow family of flowering plants in the case of *Cx. pallidothorax*. Peony yellowing-associated secovirus, previously detected only in peonies (Viridiplantae: Paeoniaceae), was an unpublished Secoviridae virus (GenBank: QNN26213.1) as of 2021. The presence of peony yellowing-associated secovirus in *Cx. pallidothorax* implies the potential acquisition of plant viruses via plant feeding. Our study demonstrated a 96.7% nucleotide identity and 97.3% amino acid identity of RdRp in peony yellowing-associated secovirus compared to that previously detected in peonies. More likely, the high sequence similarity reflected a recent historical transmission event of peony yellowing-associated secovirus between willows and peonies. Plant nectar resources are intermediate for virus spreading via a food-borne transmission pathway [46]. Because whole bodies of mosquitoes were processed for virome sequencing, the possibility of nectar-associated viruses in the guts could not be excluded. Further experimental studies are required to determine whether these viruses are nonpropagative viruses associated with blood or nectar, insect-specific RNA viruses, or pathogens, which have the potential to be transmissible in animals or plants [12].

### Role of environmental associations in shaping the virome

Our virome analysis in a mosquito species deepens the understanding of the virome composition in mosquito species across different-scale regions. In Hainan Island, we found that a large fraction of RNA viruses were persistent in the same mosquito species across 3 years and collection locations, indicating that the virome composition was largely structured by mosquito species. The virome is mainly composed of inherent vertically transmitted RNA viruses and environment-derived RNA viruses [16]. Our results demonstrated that different mosquito species are the major barrier in shaping virome composition in Hainan Island, showing consistency with the findings of previous studies [5, 9, 10]. Due to the lower complexity of their food sources and living environment, field-collected mosquitoes have a higher virome diversity than laboratory-reared mosquitoes, implicating the potential for RNA virus acquisition from the local environment [16].

The distinct virome composition across mosquito species in the shared environment of Hainan Island indicates that food source preference may be another important factor affecting virome composition. This emphasizes the important contribution of environmental food availability and mosquito-feeding behavior to shape the virome dynamics in a natural ecosystem. In a relatively independent region such as Hainan Island, the feeding preferences of different mosquito species can allow them to avoid interspecific competition via the partitioning of natural resources, including foods and habitats, thereby resulting in distinct, species-specific viromes.

The diversity of mosquito viromes in Hainan Island was significantly lower than that in the three continental regions, probably because the isolated island environment and geographic barrier hindered the spread of viruses. The three continental regions may be more effective in facilitating the long-distance dispersal of viruses [47]. In our study, the virome comparison of the same mosquito species showed that only a few RNA viruses were shared in the different regions, indicating the spatial dependence of virome composition (Fig. 6D). Although *Ae. albopictus* and *Cx. pipiens quinquefasciatus* possess diverse and abundant viromes, only a few vertically transmitted RNA viruses in Hainan Island could be detected in the three continental regions. These shared RNA viruses belonged to the species-specific core virome, which refers to a set of viruses consistently associated with a host group [16]. Feeding preference varies among mosquito species and geographical habitats [15]. Indeed, the food sources in Hainan Island exhibited dramatic differences between *Ae. albopictus* and *Cx. pipiens quinquefasciatus*. The high differentiation of viromes between mosquito species in Hainan Island showed that a large fraction of endemic RNA viruses is circulated or spread in a mosquito species in local environments. In a cross-continental context, the virome in a mosquito species may be mainly shaped by environment-related food availability. Overall, the acquisition and spread of RNA viruses in a natural ecosystem are dependent on local environmental associations and mosquito species barriers. Further investigations should explore how large the range must be for the destabilization of species-specific viromes.

## Materials and methods

### Sample collection and species identification

Mosquitoes were sampled in the summer for three consecutive years (2018 to 2020) across 13 geographic districts in Hainan Island in China, including Baisha County (BS), Dongfang City (DF), Danzhou City (DZ), Haikou City (HK), Ledong County (LD), Lingao County (LG), Lingshui County (LS), Qinghai City (QH), Sanya City (SY), Tunchang County (TC), Wenchang City (WC), Wanning City (WN), and Wuzhishan City (WZS). All mosquito samples were initially identified based on morphological characters initially, washed using PBS buffer, and then placed in RNAlater immediately. Genomic DNA was extracted from one leg of each mosquito using the HotSHOT method [48]. To further validate mosquito species, barcoding identification of the cytochrome *c* oxidase subunit I (COI) gene was performed using Sanger RT‒PCR sequencing. The resulting barcode sequences were searched against the Culicidae barcode records in the NCBI nonredundant protein database (nr) and the BOLD database (www.boldsystems.org).

### Virome construction, assembly, and vector-host confirmation

The total RNA was isolated from three mosquito individuals of each species using TRIzol Reagent (Invitrogen). The rRNA-depleted RNA was fragmented and converted into a strand-specific cDNA library using a TruSeq Stranded Total RNA Library Prep Kit (Illumina). The 150 bp paired-end sequencing was performed on an Illumina HiSeq 4000 System. The sequencing libraries of mosquito species were generally proportional to their prevalence in field estimates from two previous studies, which showed that *Ae. albopictus*, *Cx. pipiens quinquefasciatus*, and *Ar. subalbatus* were the most prevalent mosquito species in Hainan Island [17, 37]. Adaptors and low-quality bases in the raw data were filtered by Trimmomatic 0.36 [49]. De novo transcriptome assembly was performed using Trinity 2.4.0 [50] with default parameters. Transcriptomic-based host confirmation was performed based on the concatenation of multiple single-copy orthologous sequences. The single-copy orthologs identified by BUSCO v5 (insecta_odb10) were aligned using prank v.170427 software in a codon-aware nucleotide manner [51]. The ambiguously aligned regions in multiple sequence alignments were removed using the TrimAl 1.41 software [52]. The best-fitting model was determined by the ModelFinder program implemented in IQ-TREE v2.1.3 based on the Bayesian information criterion [53, 54]. Phylogenetic inference by the maximum likelihood (ML) method was performed using an SH-like approximate likelihood ratio test and ultrafast bootstrap approximation with 1000 replicates. The mosquito samples with concordant species identification results from the morphological and barcoding methods were used for subsequent analysis.

### RNA virus discovery

All assembled contigs were translated into six frames using the Transeq program embedded in the EMBOSS 6.6.0 software package [55] and were used to identify viral sequences using diamond v0.9.26 BLASTP searches [56]. The potential viral sequences had hits in at least one of the following cases: (*i*) against the virus protein database from GenBank (downloaded on 8/18/2021) with a 1e-5 value cutoff; (*ii*) against the RNA virus sequences in the Virus Metadata Repository (International Committee on Taxonomy of Viruses, ICTV; version July 20-2021) with a 1e-5 value cutoff; and (*iii*) against the RNA-dependent RNA polymerase (RdRp)-related position-specific score matrices (PSSMs) in Conserved Domain Database (CDD) version 3.19 using domain-based RPS-BLAST with a 1e−2 value cutoff. False-positive contigs were removed based on BLAST searches against three databases: the nr database, the nonredundant nucleotide database (nt), and the genome sequences within the family Culicidae. The redundant duplicates were clustered using CD-HIT-EST v4.8.1 if their nucleotide identities were higher than 90% (Fu et al. 2012). The contigs showing the best hits against the viral proteins under Riboviria (NCBI Taxid 2559587, excluding retroviruses) and whose nucleotide lengths were greater than 300 were kept for further analysis. The clustered putative viral RdRp contigs were considered different viral taxonomic units and thus could be used to explore virome community structure and diversity. The RdRp protein is considered “complete or nearly complete” if it aligns with >80% coverage of the known virus species deposited in the public databases mentioned above. Novel viruses are those in which sequences match with <80% aa identity and <90% nucleotide identity. For multiple-segment RNA viruses, segments from the same virus species were identified based on co-occurrence frequency and codon usage in different mosquito samples. We further verified the viral genome sequences provided here by mapping reads to their corresponding contigs and by assessing the continuity of read coverage manually using the IGV 2.7.2 program [57]. RT‒PCR and Sanger sequencing were then utilized to further confirm the assembly correctness of the virus genomes.

### Phylogenetic inference and RNA virome annotation

In phylogenetic inference, multiple sequence alignments of RdRp proteins were performed using MAFFT v7.215 [58], employing the E-INS-I algorithm. The methods for ambiguous site removal, model selection, and virus phylogenetic relationship inference followed the methods described in the host confirmation section. Pruning, organization, and visualization of phylogenetic trees were carried out using iTOL v6.5.8 [59]. Putative viral open reading frames (ORFs) were identified in accordance with the following standards: (i) aa length greater than 100, (ii) removing the sequences of which the aa length <200 and showing overlapping with a longer ORF, and (iii) passed a manual check with their related virus genus or family. A combination of searches including HHsearch v3.3.0 against the pdb70 database (version 220313) and RPS-BLAST search against NCBI CDD and the nr database was utilized to annotate viral ORFs [60].

### Food source identification and co-occurrence association analysis of RNA viruses

If the contigs were aligned to the genomic sequences from arthropods (NCBI Taxid 6656 and its subordinate Taxid) in the nr/nt database (latest access date, 01/10/2022), they were considered “presumptive host contigs” and thus were removed from the analyses. The remaining contigs were taxonomically assigned to the Eukaryota taxon (except for fungi) to identify the putative bloodmeal sources using BLAST searches against the nr/nt databases. Putative food sources were determined based on the following criteria: a 1e−5 value cutoff, an aligned fragment with a size greater than 200 bp, and alignment with >80% coverage of the contigs. If the contigs showed the best hit to multiple species with equivalent BLAST bitscores, they were annotated to the last common ancestor (LCA). Due to the absence of genome sequences in environments, our analysis was set to the family level and genus level. The hypergeometric test was used to determine the statistical significance of co-occurrence events between food sources and RNA viruses.

### Viral abundance and diversity

All RdRp ORFs, including those downloaded from Genebank 8/18/2021 and those from newly identified viruses in this study, clustered into divergent virus OTUs on the basis of a protein identity of 90%. Non-rRNA reads from each sample were mapped against the above virus OTUs using BBMap v38.96 (sourceforge.net/projects/bbmap/), and only the virus OTUs with read coverage >50% were considered as expressed viruses for further analysis. The viral richness of each sample was estimated using the number of viral OTUs. The virus abundance of each sample was evaluated using the total number of all viral reads per million (RPM) using the formula “total mapped viral read/total non-rRNA reads * 1,000,000”. The Shannon index (*H*) of the virome in the sequencing libraries was determined using the following formula:

$$H =-\sum_{i = 1}^{s}{p}_{i}ln{p}_{i}$$

where *S* is the number of RNA viruses identified in each sequencing library and *pi* is the proportional abundance of the *i*th RNA virus relative to the overall RNA viruses.

## Supplementary Information

**Additional file 1:** **Fig. S1.** Phylogeny of viral RdRp protein sequences in the phylum *Lenarviricota*. The red star indicates bootstrap support >90%. Branch lengths are measured by a scale bar. The RNA virus identified in this study is labeled by red branches. The red circle represents the novel virus identified in this study, and the blue circle represents the previously described virus. **Fig. S2.** Phylogeny of viral RdRp protein sequences in the family *Partitiviridae*. The red star indicates bootstrap support >90%. Branch lengths are measured by a scale bar. The RNA virus identified in this study is labeled by red branches. The red circle represents the novel virus identified in this study, and the blue circle represents the previously described virus. **Fig. S3.** Phylogeny of viral RdRp protein sequences in the family *Iflaviridae*. The red star indicates bootstrap support >90%. Branch lengths are measured by a scale bar. The RNA virus identified in this study is labeled by red branches. The red circle represents the novel virus identified in this study, and the blue circle represents the previously described virus. **Fig. S4.** Phylogeny of viral RdRp protein sequences in the family *Secoviridae*. The red star indicates bootstrap support >90%. Branch lengths are measured by a scale bar. The RNA virus identified in this study is labeled by red branches. The red circle represents the novel virus identified in this study, and the blue circle represents the previously described virus. **Fig. S5.** Phylogeny of viral RdRp protein sequences in the family *Dicistroviridae*. The red star indicates bootstrap support >90%. Branch lengths are measured by a scale bar. The RNA virus identified in this study is labeled by red branches. The red circle represents the novel virus identified in this study, and the blue circle represents the previously described virus. **Fig. S6.** Phylogeny of viral RdRp protein sequences in the family *Polycipiviridae*. The red star indicates bootstrap support >90%. Branch lengths are measured by a scale bar. The RNA virus identified in this study is labeled by red branches. The red circle represents the novel virus identified in this study, and the blue circle represents the previously described virus. **Fig. S7.** Phylogeny of viral RdRp protein sequences in the families *Picornaviridae* and *Solinviviridae*. The red star indicates bootstrap support >90%. Branch lengths are measured by a scale bar. The RNA virus identified in this study is labeled by red branches. The red circle represents the novel virus identified in this study, and the blue circle represents the previously described virus. **Fig. S8.** Phylogeny of viral RdRp protein sequences in the family *Endornaviridae*. The red star indicates bootstrap support >90%. Branch lengths are measured by a scale bar. The RNA virus identified in this study is labeled by red branches. The red circle represents the novel virus identified in this study, and the blue circle represents the previously described virus. **Fig. S9.** Phylogeny of viral RdRp protein sequences in the family *Virgaviridae*. The red star indicates bootstrap support >90%. Branch lengths are measured by a scale bar. The RNA virus identified in this study is labeled by red branches. The red circle represents the novel virus identified in this study, and the blue circle represents the previously described virus. **Fig. S10.** Phylogeny of viral RdRp protein sequences in the family *Tymoviridae*. The red star indicates bootstrap support >90%. Branch lengths are measured by a scale bar. The RNA virus identified in this study is labeled by red branches. The red circle represents the novel virus identified in this study, and the blue circle represents the previously described virus. **Fig. S11.** Phylogeny of viral RdRp protein sequences in the family *Flaviviridae*. The red star indicates bootstrap support >90%. Branch lengths are measured by a scale bar. The RNA virus identified in this study is labeled by red branches. The red circle represents the novel virus identified in this study, and the blue circle represents the previously described virus. **Fig. S12.** Phylogeny of viral RdRp protein sequences in the family *Nodaviridae*. The red star indicates bootstrap support >90%. Branch lengths are measured by a scale bar. The RNA virus identified in this study is labeled by red branches. The red circle represents the novel virus identified in this study, and the blue circle represents the previously described virus. **Fig. S13.** Phylogeny of viral RdRp protein sequences in the families *Totiviridae* and *Chrysoviridae*. The red star indicates bootstrap support >90%. Branch lengths are measured by a scale bar. The RNA virus identified in this study is labeled by red branches. The red circle represents the novel virus identified in this study, and the blue circle represents the previously described virus. **Fig. S14.** Phylogeny of viral RdRp protein sequences in the order *Reovirales*. The red star indicates bootstrap support >90%. Branch lengths are measured by a scale bar. The RNA virus identified in this study is labeled by red branches. The red circle represents the novel virus identified in this study, and the blue circle represents the previously described virus. **Fig. S15.** Phylogeny of viral RdRp protein sequences in the order *Bunyavirales*. The red star indicates bootstrap support >90%. Branch lengths are measured by a scale bar. The RNA virus identified in this study is labeled by red branches. The red circle represents the novel virus identified in this study, and the blue circle represents the previously described virus. **Fig. S16.** Phylogeny of viral RdRp protein sequences in the family *Orthomyxoviridae*. The red star indicates bootstrap support >90%. Branch lengths are measured by a scale bar. The RNA virus identified in this study is labeled by red branches. The red circle represents the novel virus identified in this study, and the blue circle represents the previously described virus. **Fig. S17.** Phylogeny of viral RdRp protein sequences in the family *Rhabdoviridae*. The red star indicates bootstrap support >90%. Branch lengths are measured by a scale bar. The RNA virus identified in this study is labeled by red branches. The red circle represents the novel virus identified in this study, and the blue circle represents the previously described virus. **Fig. S18.** Phylogeny of viral RdRp protein sequences in the family *Xinmoviridae*. The red star indicates bootstrap support >90%. Branch lengths are measured by a scale bar. The RNA virus identified in this study is labeled by red branches. The red circle represents the novel virus identified in this study, and the blue circle represents the previously described virus. **Fig. S19.** Phylogeny of viral RdRp protein sequences in the family *Birnaviridae*. The red star indicates bootstrap support >90%. Branch lengths are measured by a scale bar. The RNA virus identified in this study is labeled by red branches. The red circle represents the novel virus identified in this study, and the blue circle represents the previously described virus. **Fig. S20.** Saturation analysis of the identified viral family across sequencing samples. Ar.sub, Armigeres subalbatus; Ae.alb, *Aedes albopictus*; Ae.vex, *Aedes vexans*; Cx.gel, *Culex gelidus*; Cx.pal, *Culex pallidothorax*; Cx.pip, *Culex pipiens quinquefasciatus*; Cx.tri, *Culex tritaeniorhynchus*; Cx.vis, *Culex vishnui*. **Fig. S21.** Random validation of plant-derived RNAs and RNA viruses based on RT‒PCR Sanger sequencing. Cx.pip, *Culex pipiens quinquefasciatus*; Ae.vex, *Aedes vexans*; Cx.pal, *Culex pallidothorax*; Cx.gel, *Culex gelidus*; HMOV5, Hainan Mosquito Virus 5. **Fig. S22.** The observed richness of operational taxonomic units of RNA viruses (A) and RNA virus abundance (B) in different sequencing libraries across mosquito species. *P < 0.05; **P < 0.001. Ae.alb, *Aedes albopictus*; Ae.aeg, *Aedes aegypti*; Cx.pip, *Culex pipiens quinquefasciatus*; Ar.sub, *Armigeres subalbatus*; Cx.tri, *Culex tritaeniorhynchus*; Cx.pal, *Culex pallidothorax*; Ae.vex, *Aedes vexans*; Cx.vis, *Culex vishnui*; Cx.fus, *Culex fuscocephala*; Cx.sit, *Culex sitiens*; Cx.pse, *Culex pseudovishnui*; An.vag, *Anopheles vagus*; Cx.lut, *Culex (Lutzia) fuscanus*; Ae.mal, *Aedes malayensis*; Cx.gel, *Culex gelidus*. **Fig. S23.** Multiple scaling analysis for RNA virome composition across mosquito species using a Euclidean distance matrix. BS, Baisha. DF, Dongfang. DZ, Danzhou. HK, Haikou. LD, Ledong. LG, Lingao. LS, Lingshui. QH, Qionghai. SY, Sanya. TC, Tunchang. WC, Wenchang. WN, Wanning. WZS, Wuzhishan. **Fig. S24.** Heatmap plot showing the presence of virus OTUs over the three consecutive years from 2018 to 2020 in three mosquito species. Ae.alb, *Aedes albopictus*; Ar.sub, *Armigeres subalbatus*; Cx.pip, *Culex pipiens quinquefasciatus*. **Fig. S25.** Shannon index of operational taxonomic units of RNA viruses across collection locations. Statistically significant differences were detected using a Kruskal‒Wallis test in cases of multiple comparisons. In pairwise comparisons, significant differences were detected by pairwise Wilcoxon rank sum tests. *P < 0.05; **P < 0.001. BS, Baisha; DF, Dongfang; DZ, Danzhou; HK, Haikou; LD, Ledong; LG, LinGao; LS, Lingshui; QH, Qionghai; SY, Sanya; TC, Tunchang; WC, Wenchang; WN, Wanning; WZS, Wuzhishan. **Fig. S26.** The observed richness of operational taxonomic units of RNA viruses (A, C, E) and RNA virus abundance (B, D, F) between Hainan Island and other collection locations in Yunnan (inland China, Asia), California (United States, North America) and Sweden (Europe). In pairwise comparisons, Wilcoxon rank sum tests were applied to detect significant differences. *P < 0.05; **P < 0.01. Ae.alb, *Aedes albopictus*; Cx.pip, *Culex pipiens quinquefasciatus*. **Fig. S27.** Bubble plot showing the distribution of food sources derived from animals and plants in *Aedes albopictus* and *Culex pipiens quinquefasciatus *in Hainan Island. The shared families are linked using blue lines. Ae.alb, *Aedes albopictus*;Cx.pip, *Culex pipiens quinquefasciatus*. **Fig. S28.** Bubble plot showing the distribution of food sources derived from animals and plants in *Aedes albopictus* (left) and *Culex pipiens quinquefasciatus* (right) in the four continental regions. The shared families are linked using blue lines.

**Additional file 2:** **Table S1.** Sequencing statistics of mosquito samples in this study.

**Additional file 3:** **Table S2.** Summary of the RNA viruses detected in this study.

**Additional file 4:** **Table S3.** Number of RNA viruses containing full-length RdRps detected in each mosquito species.

**Additional file 5:** **Table S4.** Virome sequencing data retrieved from the NCBI database.

## Data Availability

The virome data were deposited in Science Data Bank (ScienceDB) Database under the DOI accession link http://www.doi.org/10.57760/sciencedb.06132.
