# Supplementary material for: Association of virome dynamics with mosquito species and environmental factors
Source: Microbiome. 2023 May 8;11:101. doi: 10.1186/s40168-023-01556-4 (PMC10165777; doi:10.1186/s40168-023-01556-4)
Supplement: Supplementary file 2 — Additional file 1: Fig. S1. Phylogeny of viral RdRp protein sequences in the phylum Lenarviricota. The red star indicates bootstrap support >90%. Branch lengths are measured by a scale bar. The RNA virus identified in this study is labeled by red branches. The red circle represents the novel virus identified in this study, and the blue circle represents the previously described virus. Fig. S2. Phylogeny of viral RdRp protein sequences in the family Partitiviridae. The red star indicates bootstrap support >90%. Branch lengths are measured by a scale bar. The RNA virus identified in this study is labeled by red branches. The red circle represents the novel virus identified in this study, and the blue circle represents the previously described virus. Fig. S3. Phylogeny of viral RdRp protein sequences in the family Iflaviridae. The red star indicates bootstrap support >90%. Branch lengths are measured by a scale bar. The RNA virus identified in this study is labeled by red branches. The red circle represents the novel virus identified in this study, and the blue circle represents the previously described virus. Fig. S4. Phylogeny of viral RdRp protein sequences in the family Secoviridae. The red star indicates bootstrap support >90%. Branch lengths are measured by a scale bar. The RNA virus identified in this study is labeled by red branches. The red circle represents the novel virus identified in this study, and the blue circle represents the previously described virus. Fig. S5. Phylogeny of viral RdRp protein sequences in the family Dicistroviridae. The red star indicates bootstrap support >90%. Branch lengths are measured by a scale bar. The RNA virus identified in this study is labeled by red branches. The red circle represents the novel virus identified in this study, and the blue circle represents the previously described virus. Fig. S6. Phylogeny of viral RdRp protein sequences in the family Polycipiviridae. The red star indicates bootstrap support >90%. Branch l [file 40168_2023_1556_MOESM1_ESM.docx]

Supplementary Materials for

# Association of virome dynamics with mosquito species and environmental factors

Qing Liu^1, 3, 4ǂ^, Feng Cui^3ǂ^, Xiang Liu^3^, Yumei Fu^2, 3^, Wenjing Fang^1^, Xun Kang^2^, Hong Lu^3^, Siping Li^2^, Biao Liu^2^, Wei Guo^3^, Qianfeng Xia^2*^, Le Kang^1, 3, 4*^, Feng Jiang^1, 4*^

^1^Beijing Institutes of Life Science, Chinese Academy of Sciences, Beijing, China

^2^Laboratory of Tropical Biomedicine and Biotechnology, School of Tropical Medicine and Laboratory Medicine, Hainan Medical University, Haikou, China

^3^State Key Laboratory of Integrated Management of Pest Insects and Rodents, Institute of Zoology, Chinese Academy of Sciences, Beijing, China

^4^CAS Center for Excellence in Biotic Interactions, University of Chinese Academy of Sciences, Beijing, China

^ǂ^ These authors have contributed equally to this work

***Corresponding:**

Le Kang, lkang@ioz.ac.cn

Feng Jiang, jiangf@biols.ac.cn

Qianfeng Xia, xiaqianfeng@hainmc.edu.cn

# Supplemental Figures


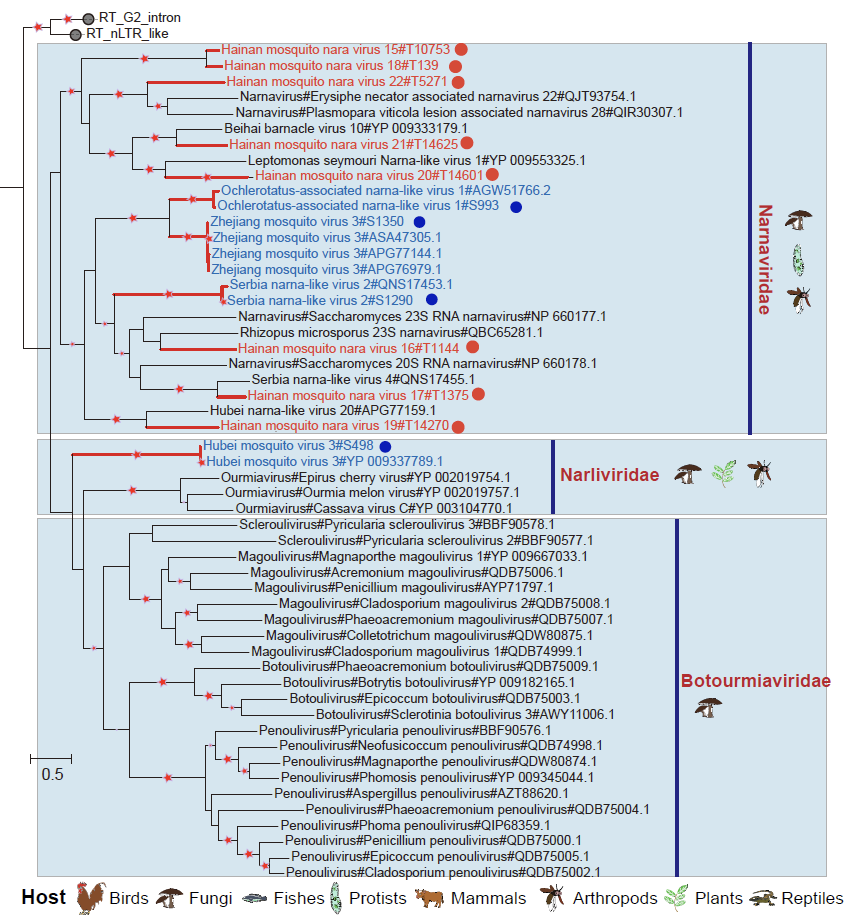


## Supplementary Figure 1. Phylogeny of viral RdRp protein sequences in the phylum *Lenarviricota*. The red star indicates bootstrap support >90%. Branch lengths are measured by a scale bar. The RNA virus identified in this study is labeled by red branches. The red circle represents the novel virus identified in this study, and the blue circle represents the previously described virus.


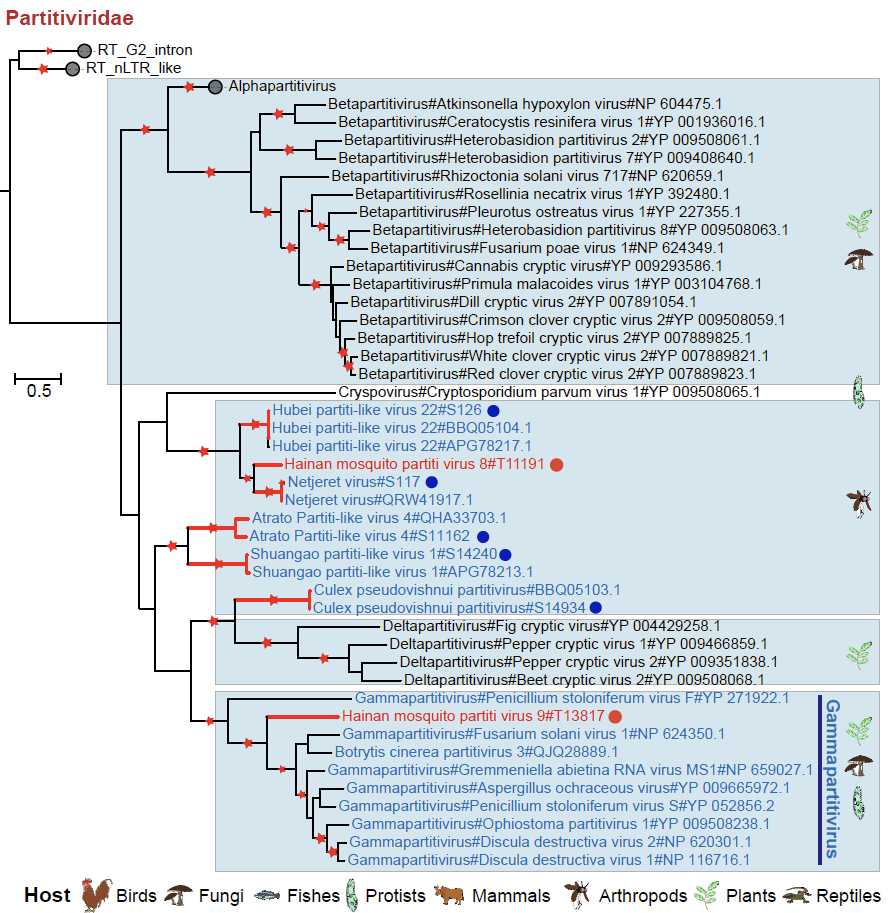


## Supplementary Figure 2. Phylogeny of viral RdRp protein sequences in the family *Partitiviridae*. The red star indicates bootstrap support >90%. Branch lengths are measured by a scale bar. The RNA virus identified in this study is labeled by red branches. The red circle represents the novel virus identified in this study, and the blue circle represents the previously described virus.


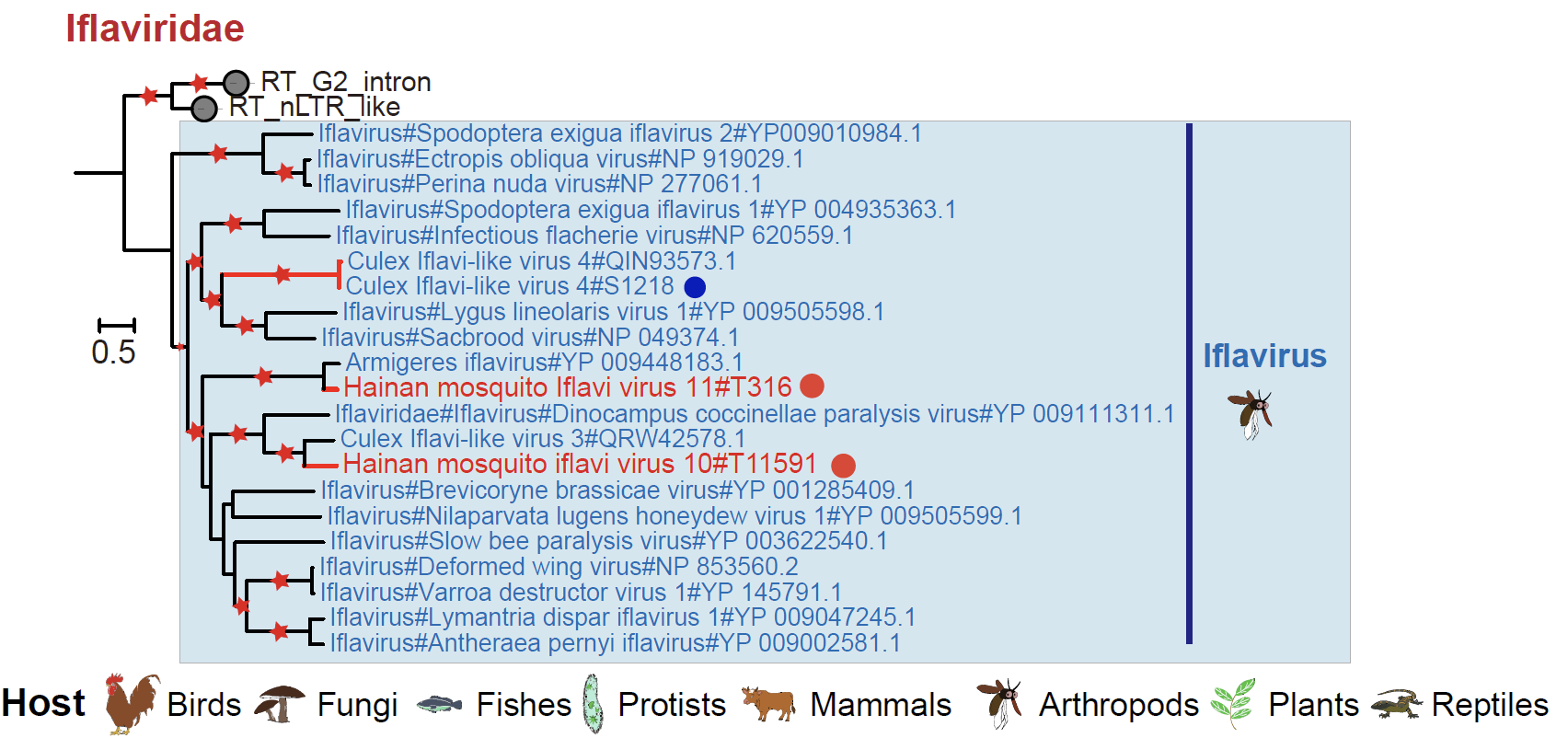


## Supplementary Figure 3. Phylogeny of viral RdRp protein sequences in the family *Iflaviridae*. The red star indicates bootstrap support >90%. Branch lengths are measured by a scale bar. The RNA virus identified in this study is labeled by red branches. The red circle represents the novel virus identified in this study, and the blue circle represents the previously described virus.


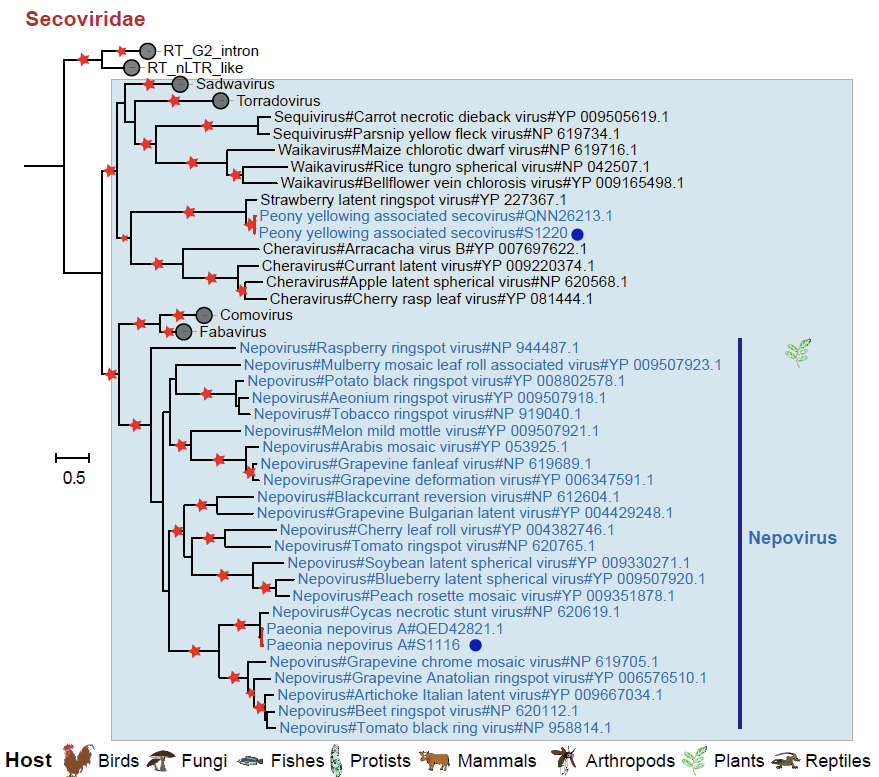


## Supplementary Figure 4. Phylogeny of viral RdRp protein sequences in the family *Secoviridae*. The red star indicates bootstrap support >90%. Branch lengths are measured by a scale bar. The RNA virus identified in this study is labeled by red branches. The red circle represents the novel virus identified in this study, and the blue circle represents the previously described virus.


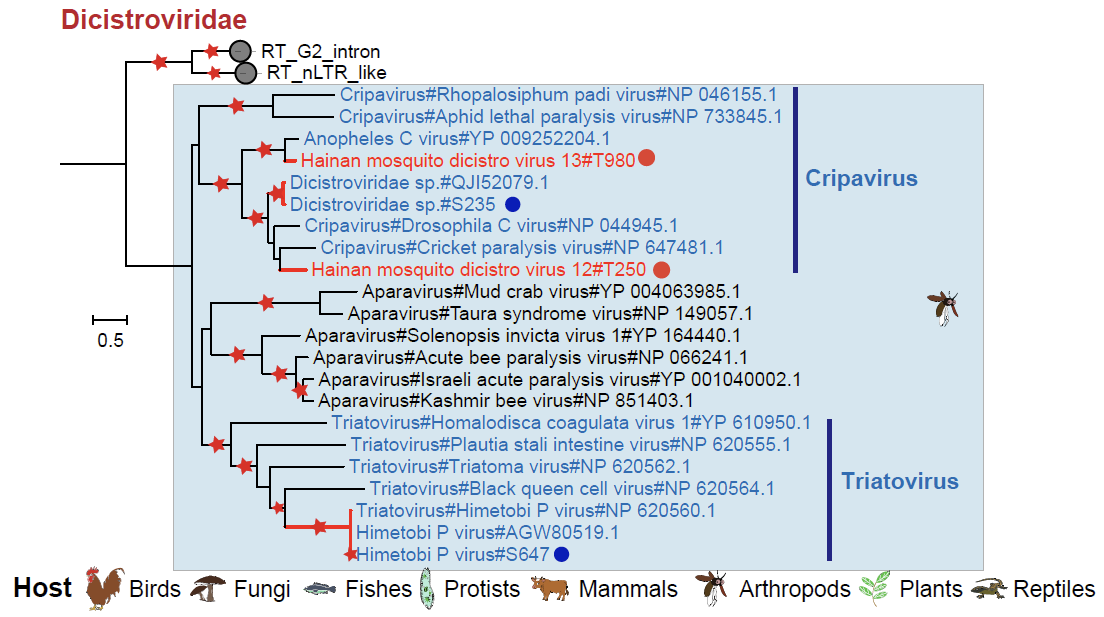


## Supplementary Figure 5. Phylogeny of viral RdRp protein sequences in the family *Dicistroviridae*. The red star indicates bootstrap support >90%. Branch lengths are measured by a scale bar. The RNA virus identified in this study is labeled by red branches. The red circle represents the novel virus identified in this study, and the blue circle represents the previously described virus.


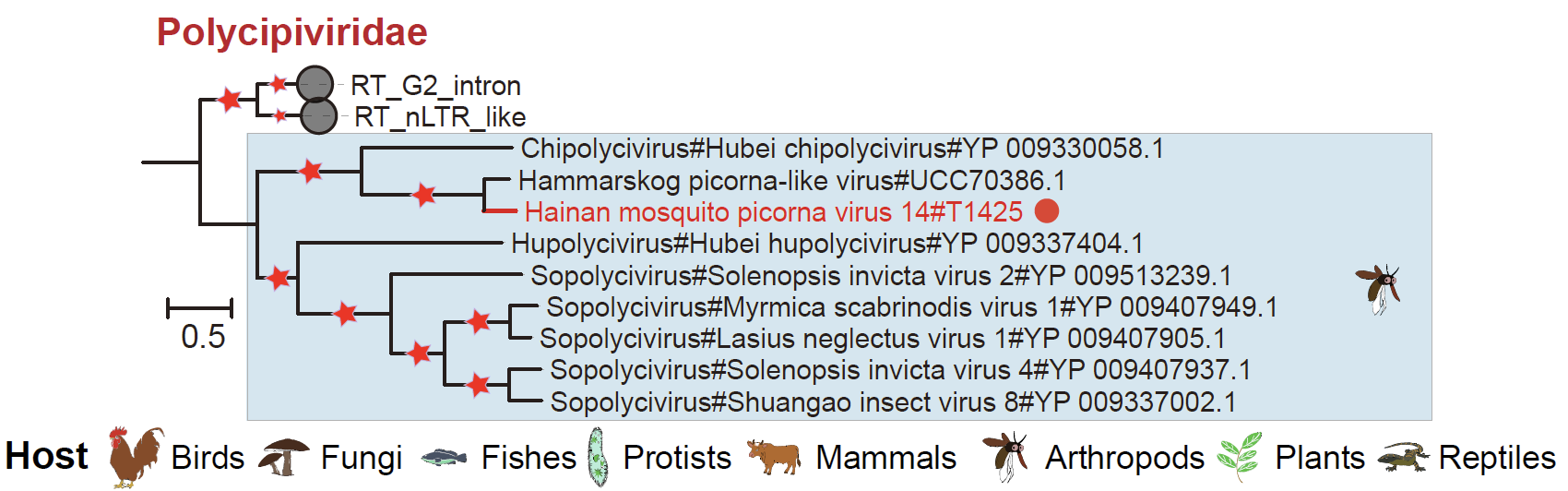


## Supplementary Figure 6. Phylogeny of viral RdRp protein sequences in the family *Polycipiviridae*. The red star indicates bootstrap support >90%. Branch lengths are measured by a scale bar. The RNA virus identified in this study is labeled by red branches. The red circle represents the novel virus identified in this study, and the blue circle represents the previously described virus.


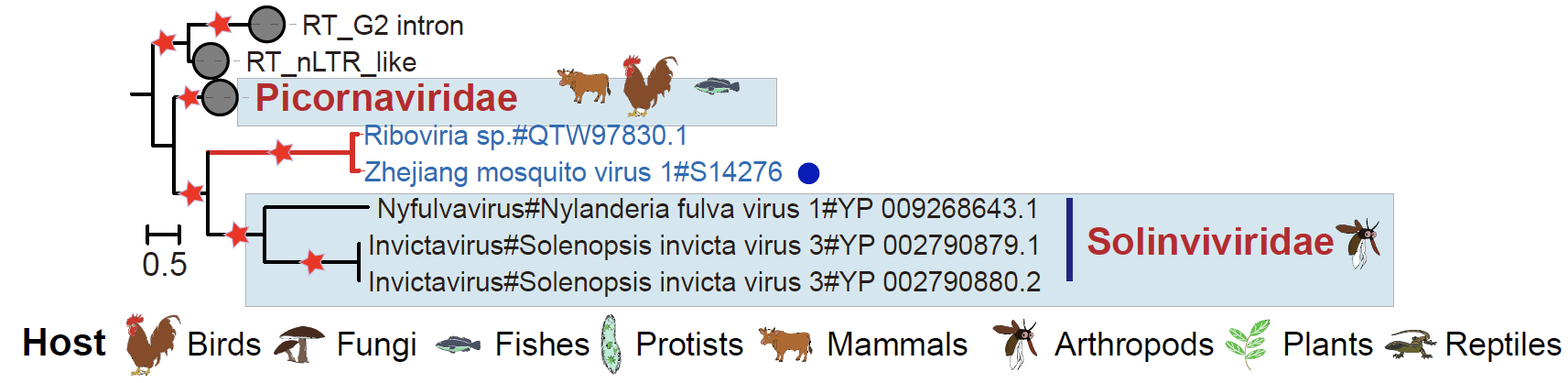


## Supplementary Figure 7. Phylogeny of viral RdRp protein sequences in the families *Picornaviridae* and *Solinviviridae*. The red star indicates bootstrap support >90%. Branch lengths are measured by a scale bar. The RNA virus identified in this study is labeled by red branches. The red circle represents the novel virus identified in this study, and the blue circle represents the previously described virus.


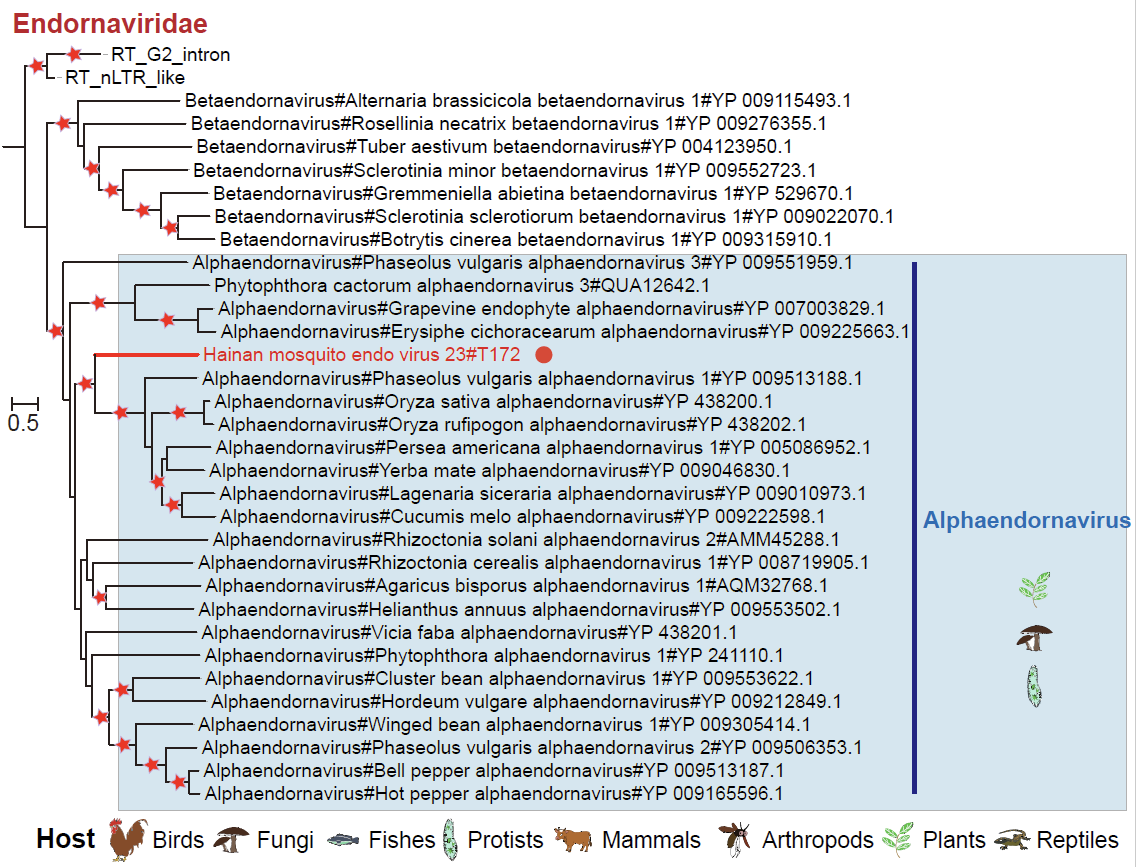


## Supplementary Figure 8. Phylogeny of viral RdRp protein sequences in the family *Endornaviridae*. The red star indicates bootstrap support >90%. Branch lengths are measured by a scale bar. The RNA virus identified in this study is labeled by red branches. The red circle represents the novel virus identified in this study, and the blue circle represents the previously described virus.


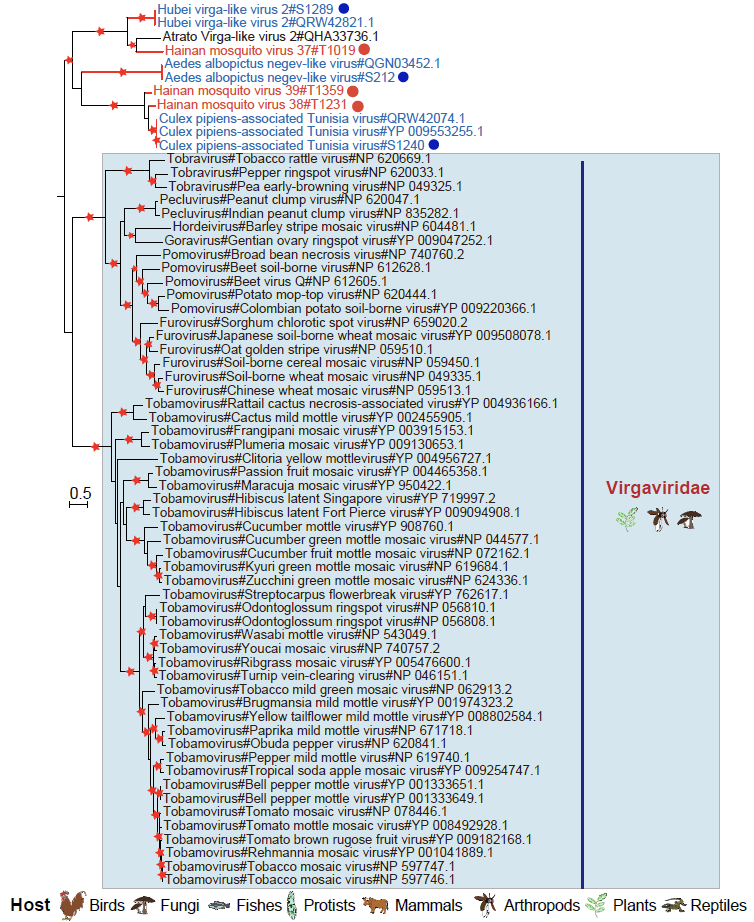


## Supplementary Figure 9. Phylogeny of viral RdRp protein sequences in the family *Virgaviridae*. The red star indicates bootstrap support >90%. Branch lengths are measured by a scale bar. The RNA virus identified in this study is labeled by red branches. The red circle represents the novel virus identified in this study, and the blue circle represents the previously described virus.


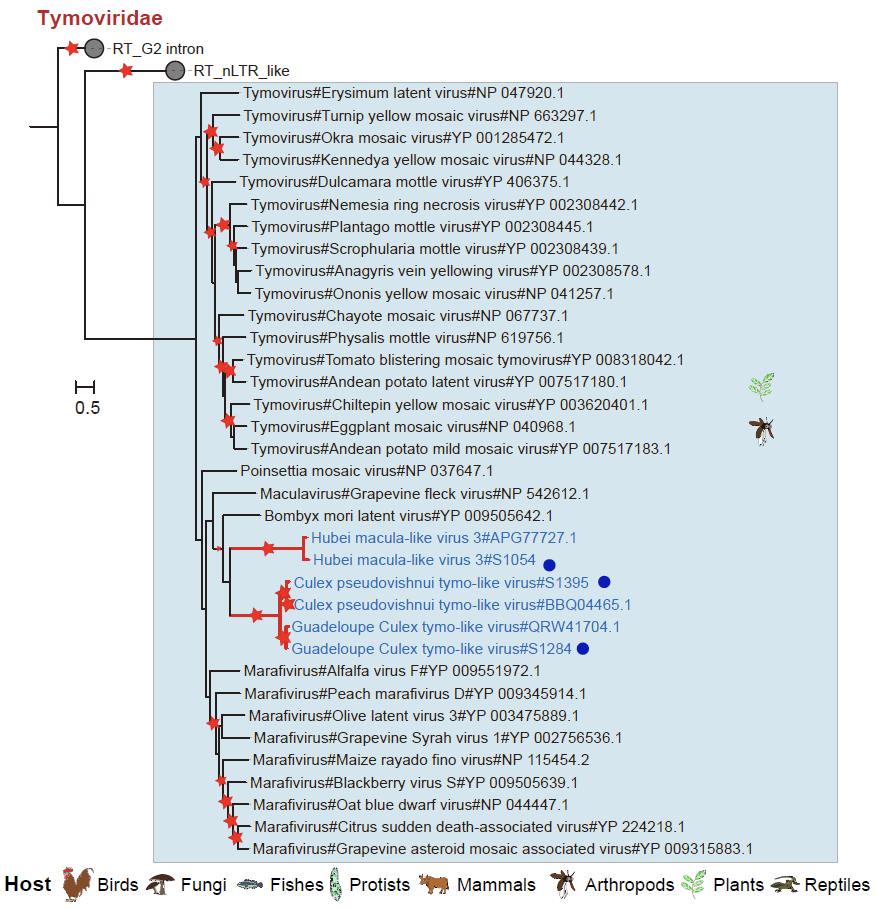


## Supplementary Figure 10. Phylogeny of viral RdRp protein sequences in the family *Tymoviridae*. The red star indicates bootstrap support >90%. Branch lengths are measured by a scale bar. The RNA virus identified in this study is labeled by red branches. The red circle represents the novel virus identified in this study, and the blue circle represents the previously described virus.


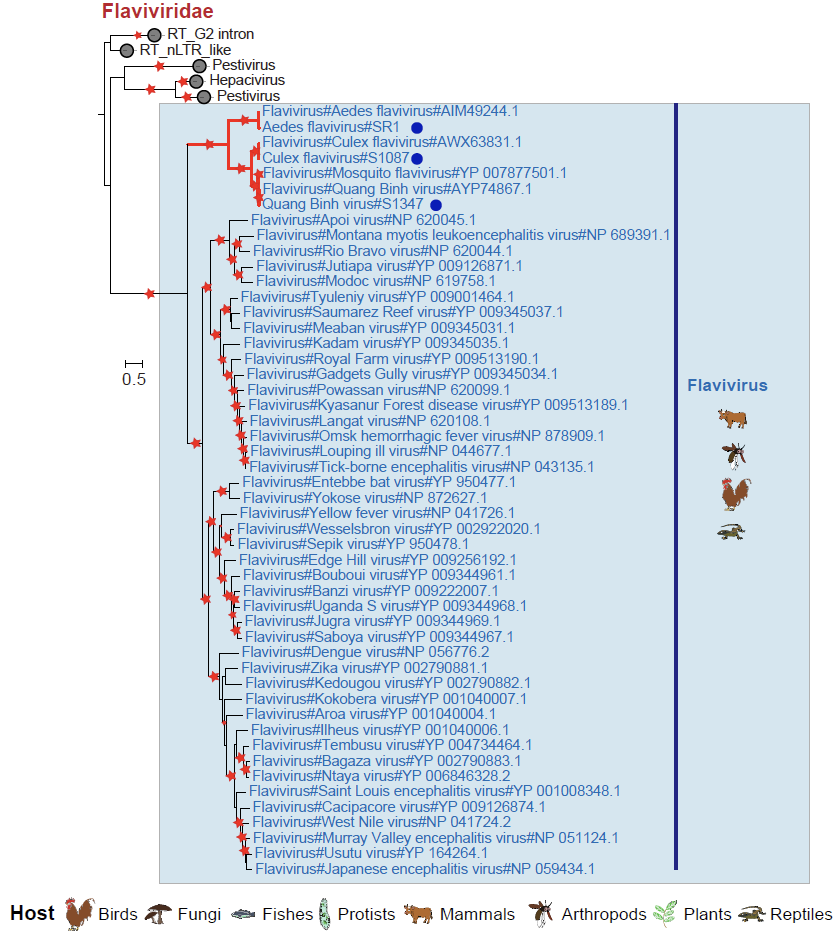


## Supplementary Figure 11. Phylogeny of viral RdRp protein sequences in the family *Flaviviridae*. The red star indicates bootstrap support >90%. Branch lengths are measured by a scale bar. The RNA virus identified in this study is labeled by red branches. The red circle represents the novel virus identified in this study, and the blue circle represents the previously described virus.


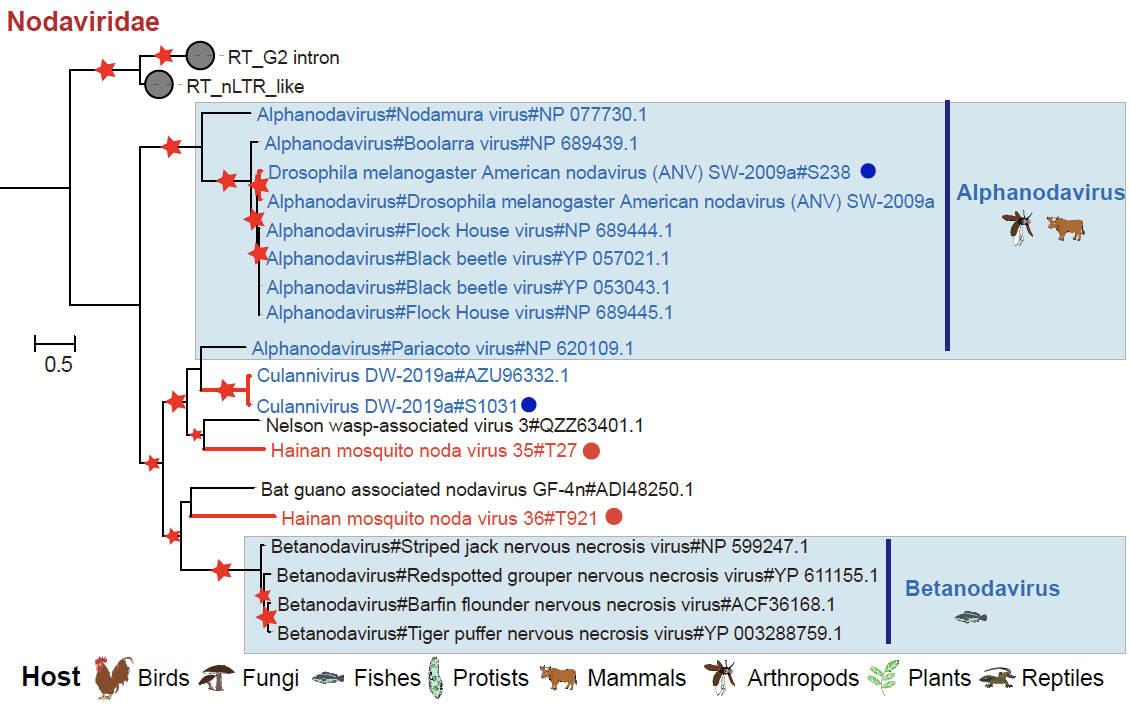


## Supplementary Figure 12. Phylogeny of viral RdRp protein sequences in the family *Nodaviridae*. The red star indicates bootstrap support >90%. Branch lengths are measured by a scale bar. The RNA virus identified in this study is labeled by red branches. The red circle represents the novel virus identified in this study, and the blue circle represents the previously described virus.


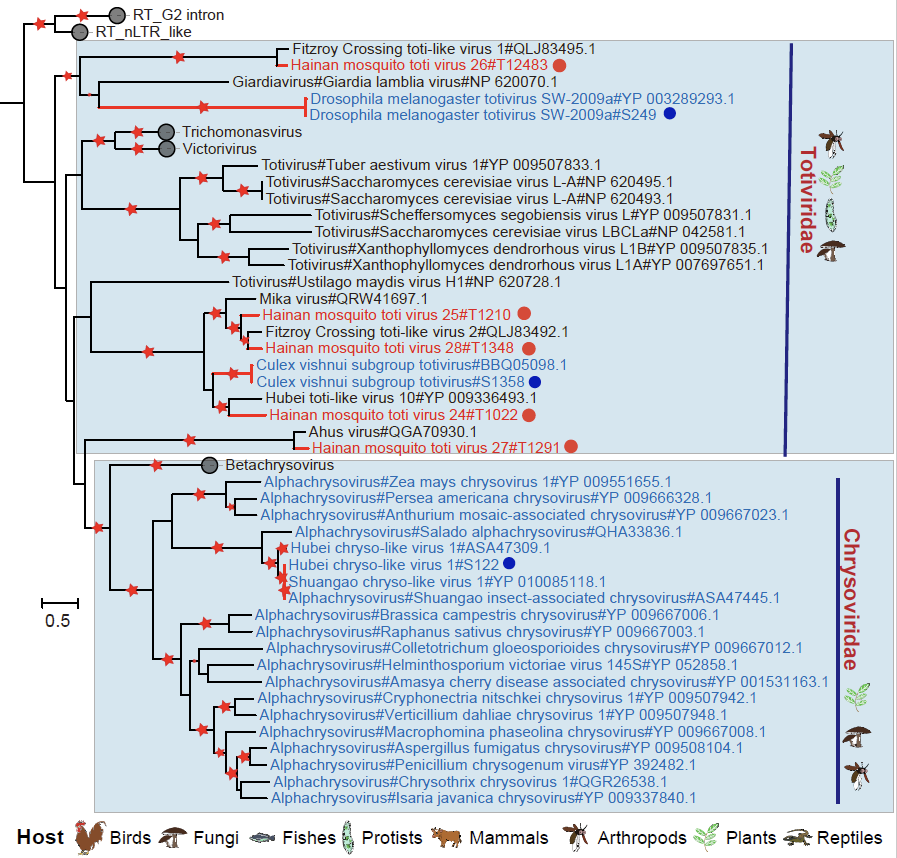


## Supplementary Figure 13. Phylogeny of viral RdRp protein sequences in the families *Totiviridae* and *Chrysoviridae*. The red star indicates bootstrap support >90%. Branch lengths are measured by a scale bar. The RNA virus identified in this study is labeled by red branches. The red circle represents the novel virus identified in this study, and the blue circle represents the previously described virus.


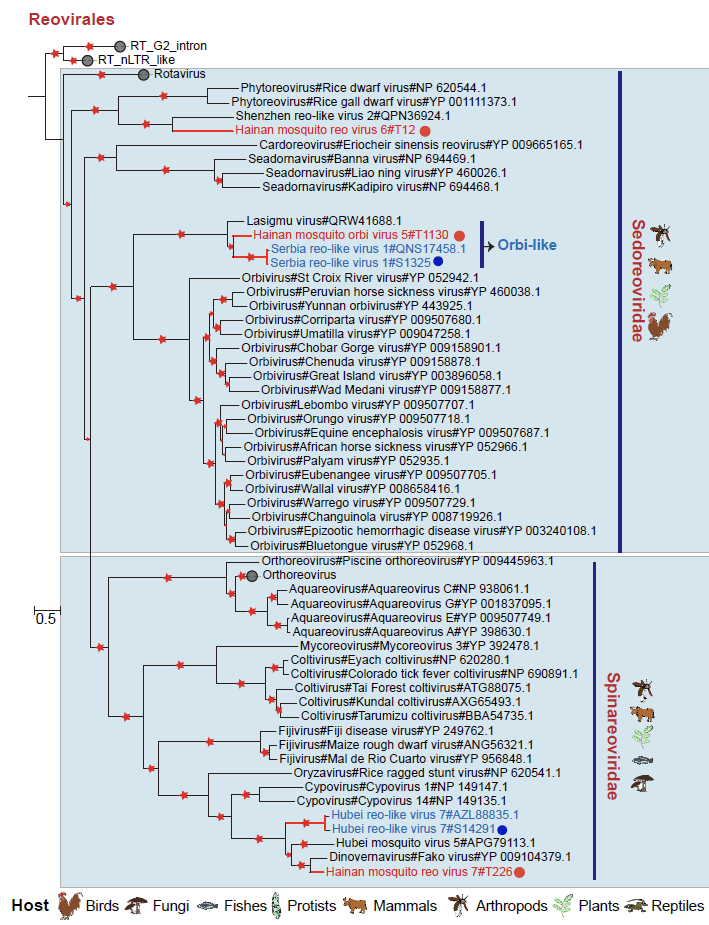


## Supplementary Figure 14. Phylogeny of viral RdRp protein sequences in the order *Reovirales*. The red star indicates bootstrap support >90%. Branch lengths are measured by a scale bar. The RNA virus identified in this study is labeled by red branches. The red circle represents the novel virus identified in this study, and the blue circle represents the previously described virus.


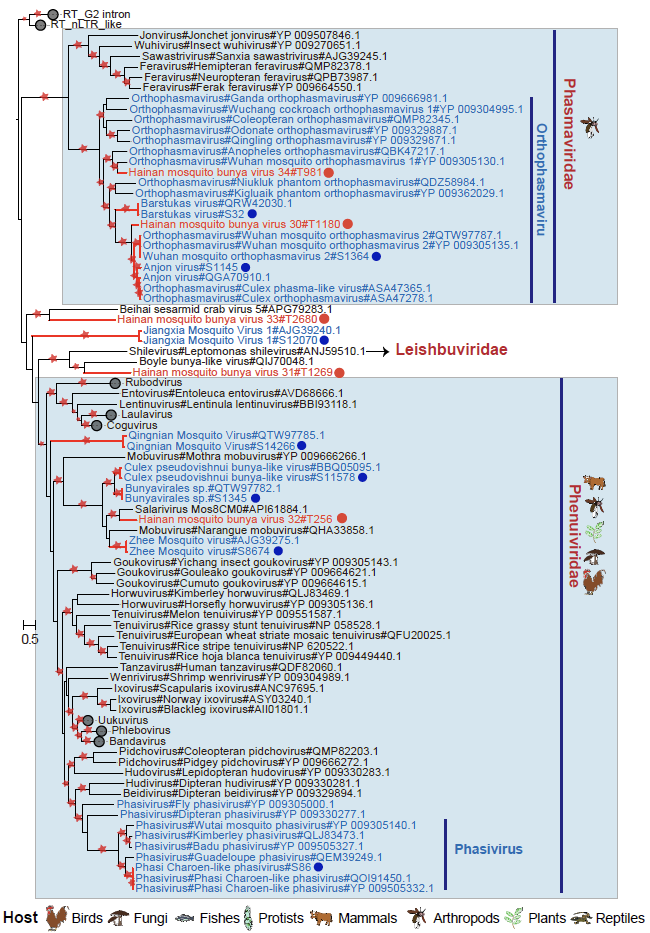


## Supplementary Figure 15. Phylogeny of viral RdRp protein sequences in the order *Bunyavirales*. The red star indicates bootstrap support >90%. Branch lengths are measured by a scale bar. The RNA virus identified in this study is labeled by red branches. The red circle represents the novel virus identified in this study, and the blue circle represents the previously described virus.


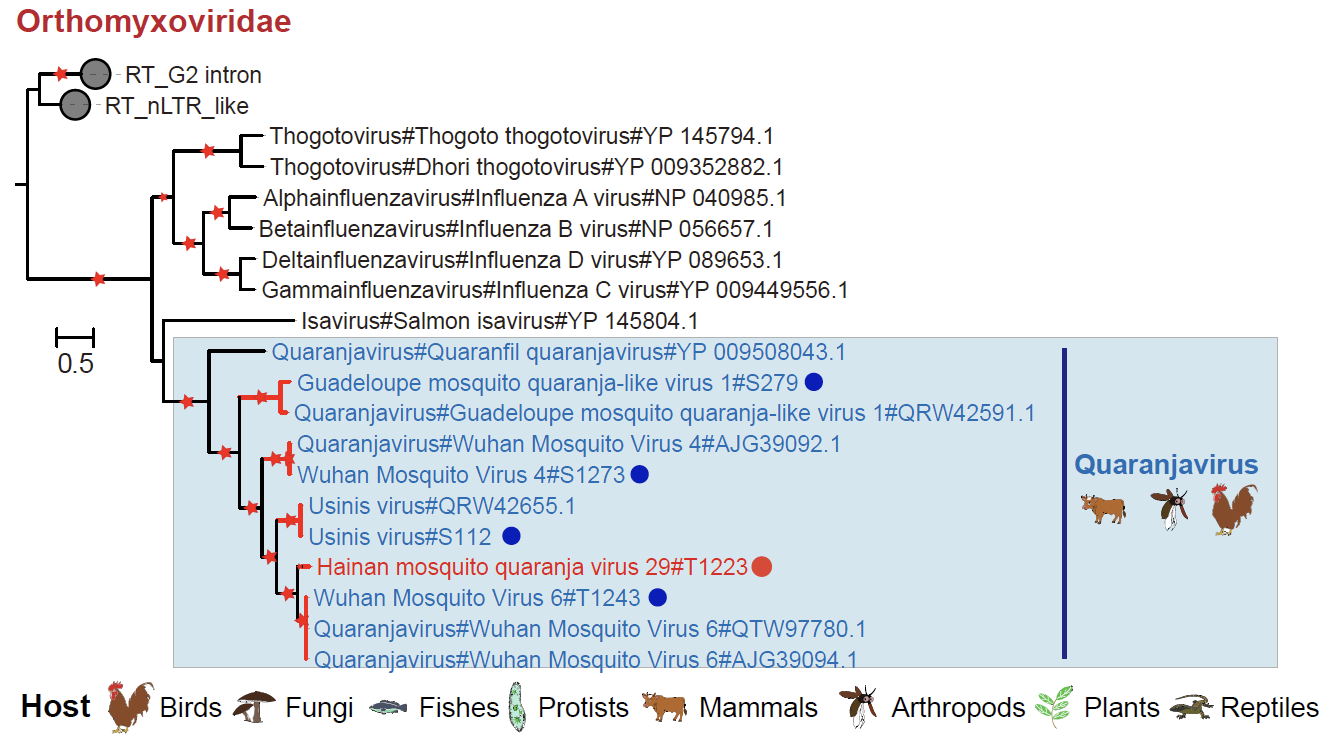


## Supplementary Figure 16. Phylogeny of viral RdRp protein sequences in the family *Orthomyxoviridae*. The red star indicates bootstrap support >90%. Branch lengths are measured by a scale bar. The RNA virus identified in this study is labeled by red branches. The red circle represents the novel virus identified in this study, and the blue circle represents the previously described virus.


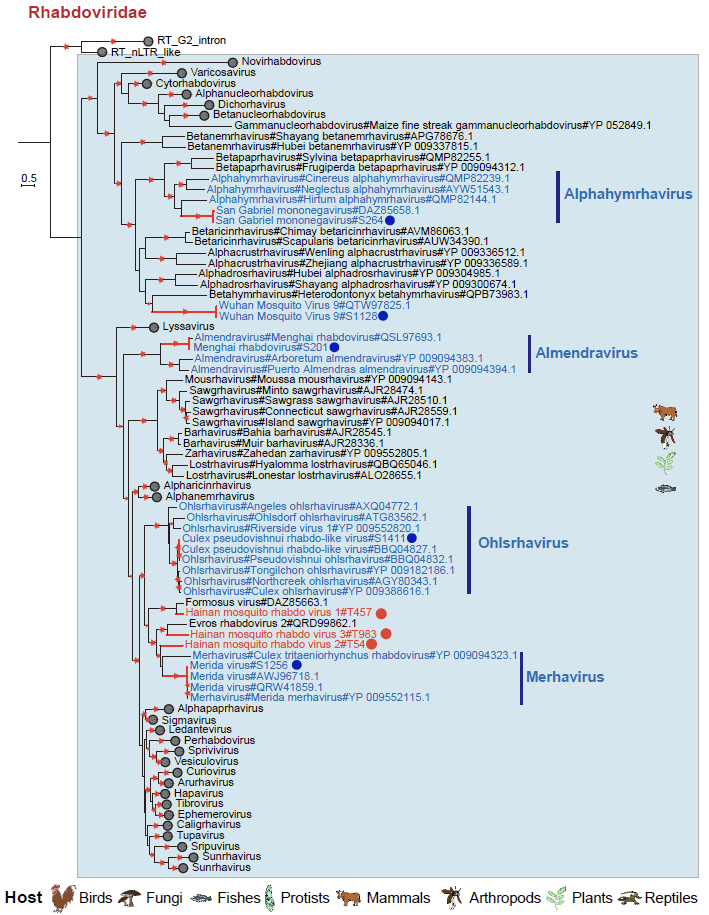


## Supplementary Figure 17. Phylogeny of viral RdRp protein sequences in the family *Rhabdoviridae*. The red star indicates bootstrap support >90%. Branch lengths are measured by a scale bar. The RNA virus identified in this study is labeled by red branches. The red circle represents the novel virus identified in this study, and the blue circle represents the previously described virus.


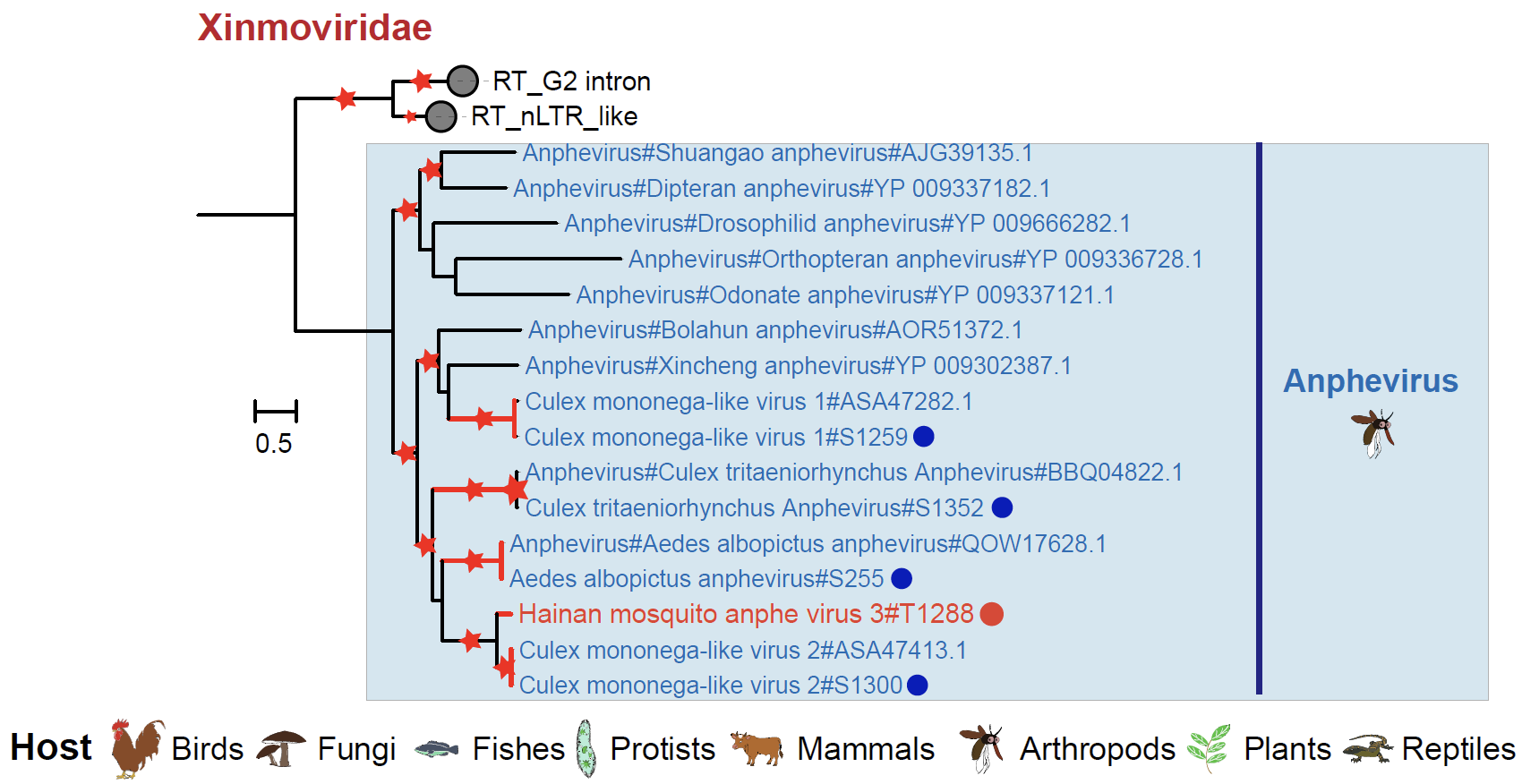


## Supplementary Figure 18. Phylogeny of viral RdRp protein sequences in the family *Xinmoviridae*. The red star indicates bootstrap support >90%. Branch lengths are measured by a scale bar. The RNA virus identified in this study is labeled by red branches. The red circle represents the novel virus identified in this study, and the blue circle represents the previously described virus.


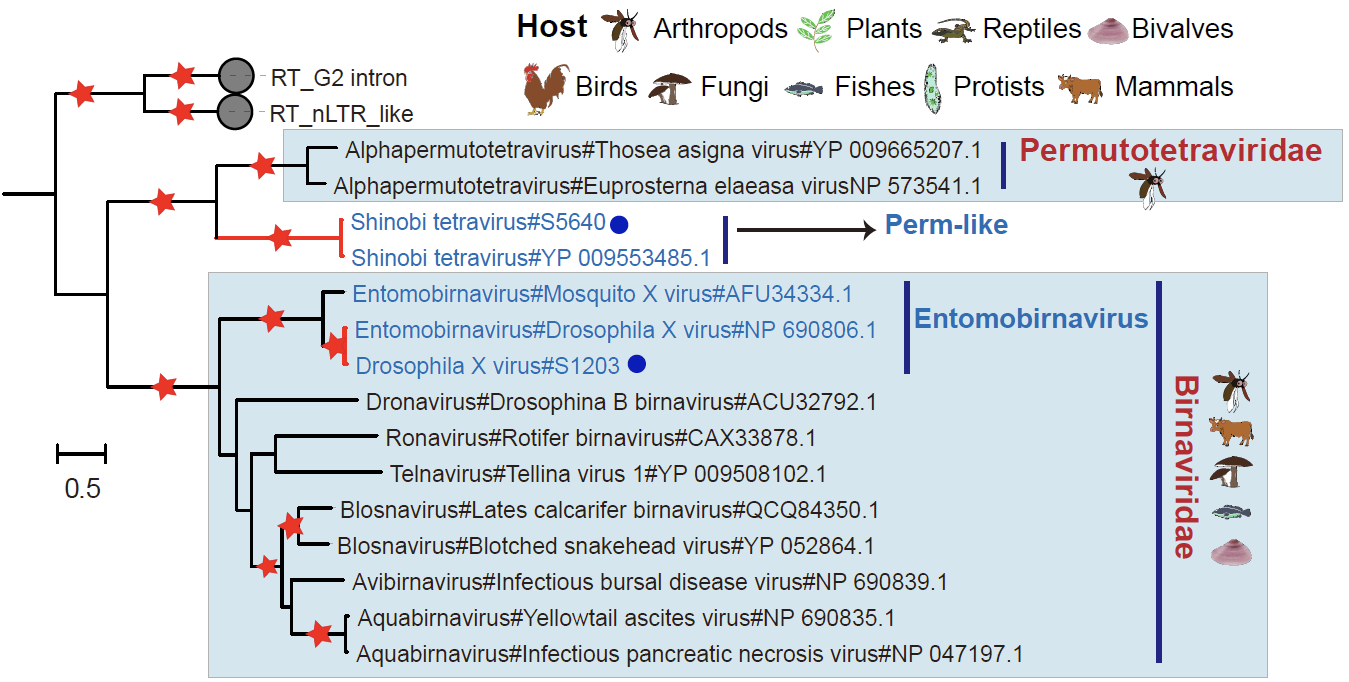


## Supplementary Figure 19. Phylogeny of viral RdRp protein sequences in the family *Birnaviridae*. The red star indicates bootstrap support >90%. Branch lengths are measured by a scale bar. The RNA virus identified in this study is labeled by red branches. The red circle represents the novel virus identified in this study, and the blue circle represents the previously described virus.


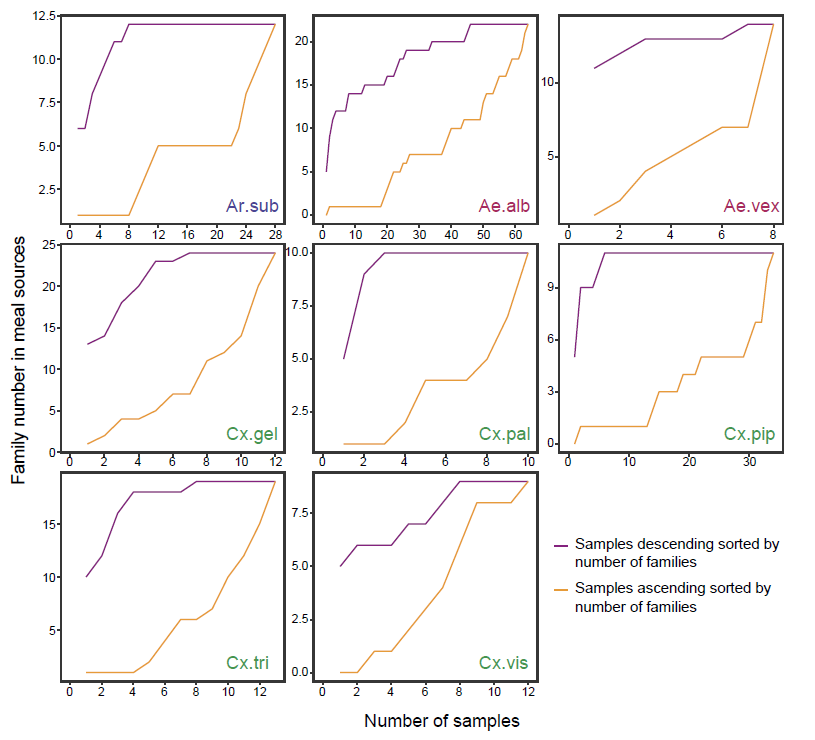


## Supplementary Figure 20. Saturation analysis of the identified viral family across sequencing samples. Ar.sub, Armigeres subalbatus; Ae.alb, *Aedes albopictus*; Ae.vex, *Aedes vexans*; Cx.gel, *Culex gelidus*; Cx.pal, *Culex pallidothorax*; Cx.pip, *Culex pipiens quinquefasciatus*; Cx.tri, *Culex tritaeniorhynchus*; Cx.vis, *Culex vishnui*.


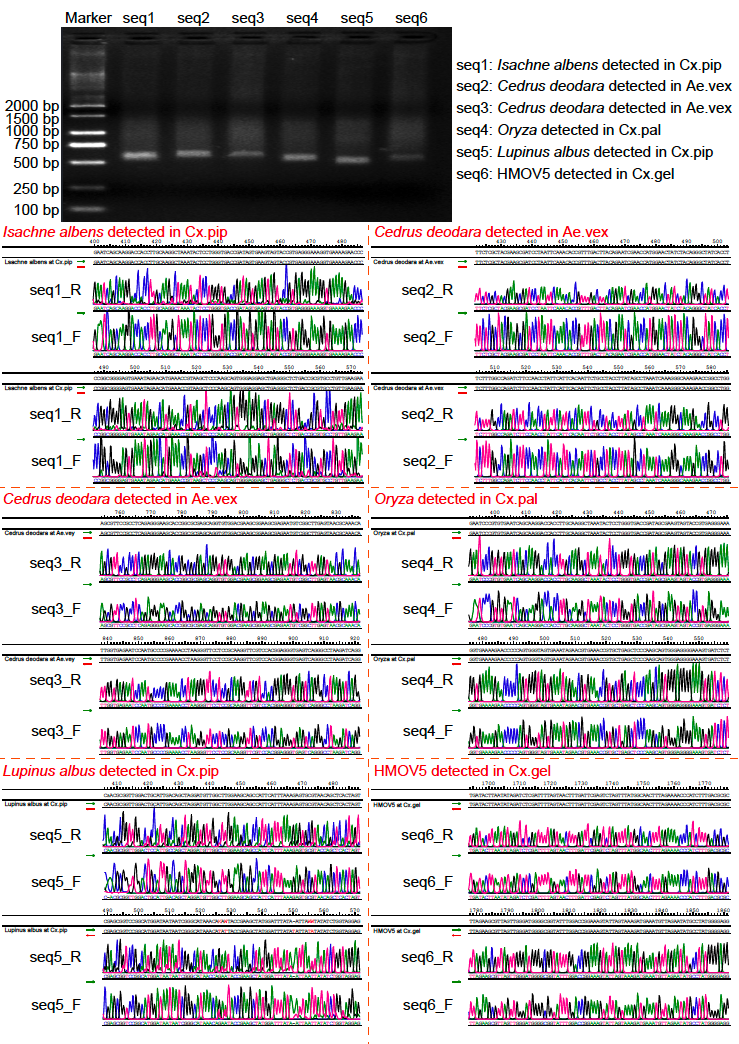


## Supplementary Figure 21. Random validation of plant-derived RNAs and RNA viruses based on RT‒PCR Sanger sequencing. Cx.pip, *Culex pipiens quinquefasciatus*; Ae.vex, *Aedes vexans*; Cx.pal, *Culex pallidothorax*; Cx.gel, *Culex gelidus*; HMOV5, Hainan Mosquito Virus 5.


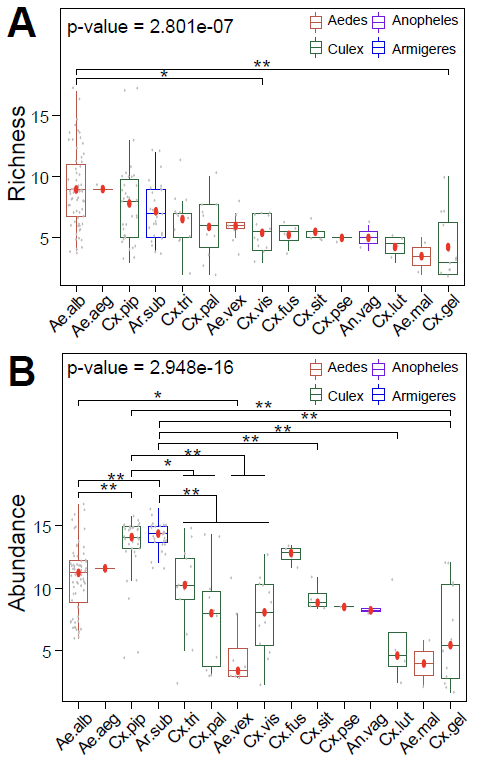


## Supplementary Figure 22. The observed richness of operational taxonomic units of RNA viruses (A) and RNA virus abundance (B) in different sequencing libraries across mosquito species. **P* < 0.05; ***P* < 0.001. Ae.alb, *Aedes albopictus*; Ae.aeg, *Aedes aegypti*; Cx.pip, *Culex pipiens quinquefasciatus*; Ar.sub, *Armigeres subalbatus*; Cx.tri, *Culex tritaeniorhynchus*; Cx.pal, *Culex pallidothorax*; Ae.vex, *Aedes vexans*; Cx.vis, *Culex vishnui*; Cx.fus, *Culex fuscocephala*; Cx.sit, *Culex sitiens*; Cx.pse, *Culex pseudovishnui*; An.vag, *Anopheles vagus*; Cx.lut, *Culex (Lutzia) fuscanus*; Ae.mal, *Aedes malayensis*; Cx.gel, *Culex gelidus*.


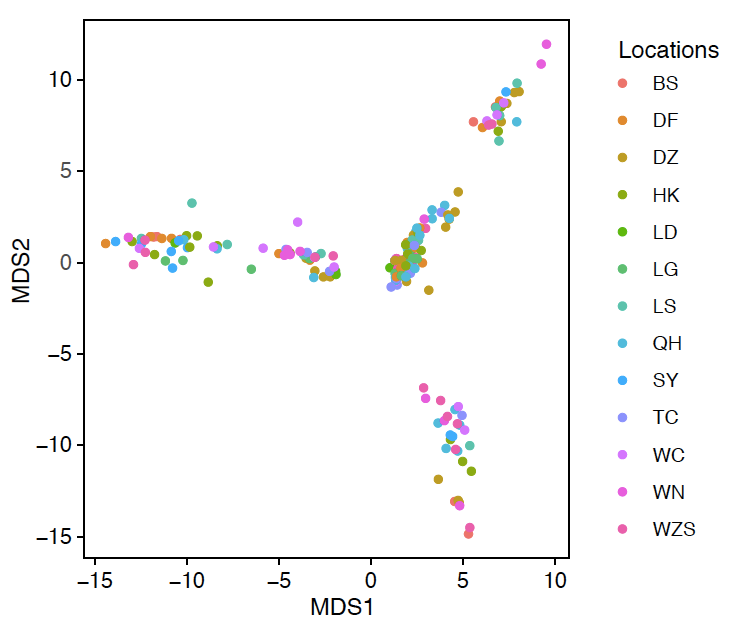


## Supplementary Figure 23. Multiple scaling analysis for RNA virome composition across mosquito species using a Euclidean distance matrix. BS, Baisha. DF, Dongfang. DZ, Danzhou. HK, Haikou. LD, Ledong. LG, Lingao. LS, Lingshui. QH, Qionghai. SY, Sanya. TC, Tunchang. WC, Wenchang. WN, Wanning. WZS, Wuzhishan.


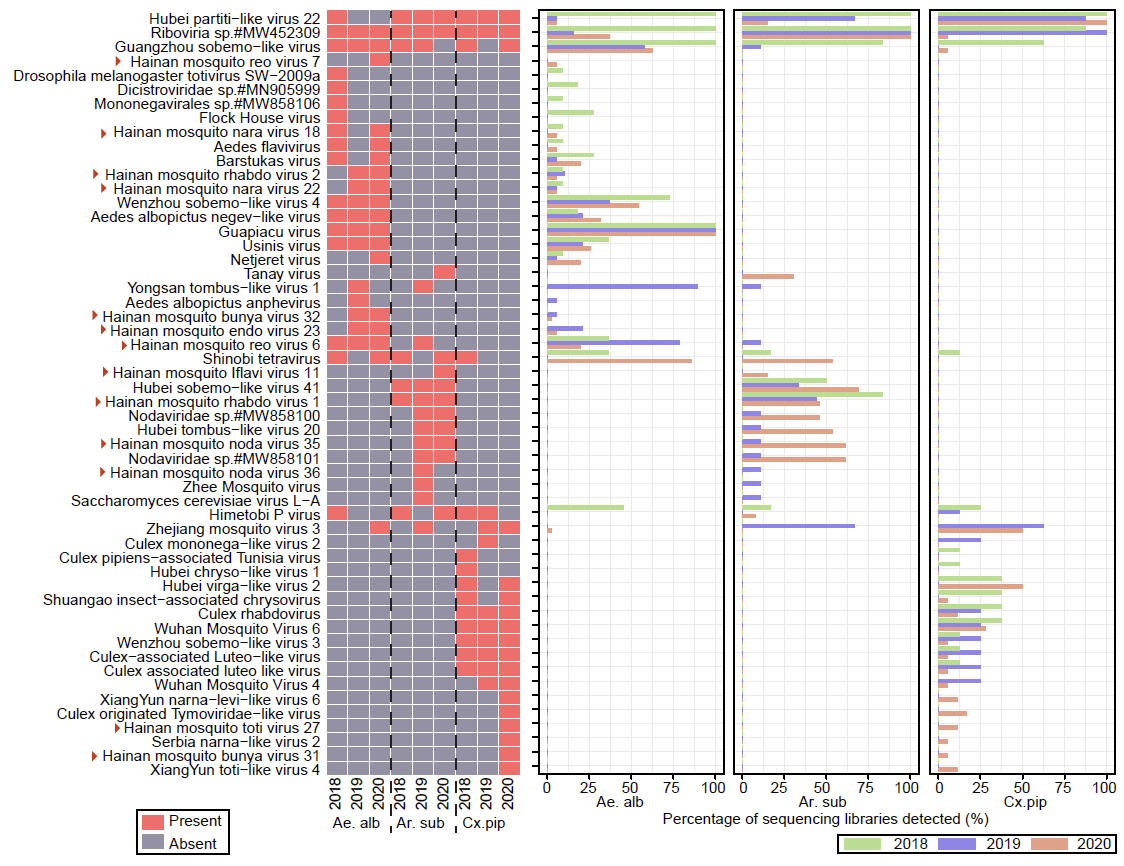


## Supplementary Figure 24. Heatmap plot showing the presence of virus OTUs over the three consecutive years from 2018 to 2020 in three mosquito species. Ae.alb, *Aedes albopictus*; Ar.sub, *Armigeres subalbatus*; Cx.pip, *Culex pipiens quinquefasciatus*.


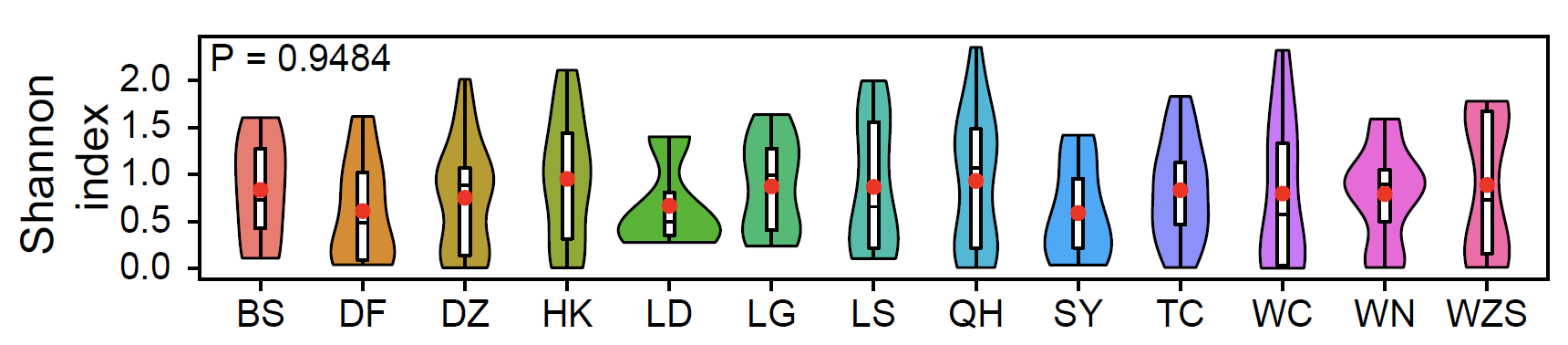


## Supplementary Figure 25. Shannon index of operational taxonomic units of RNA viruses across collection locations. Statistically significant differences were detected using a Kruskal‒Wallis test in cases of multiple comparisons. In pairwise comparisons, significant differences were detected by pairwise Wilcoxon rank sum tests. **P* < 0.05; ***P* < 0.001. BS, Baisha; DF, Dongfang; DZ, Danzhou; HK, Haikou; LD, Ledong; LG, LinGao; LS, Lingshui; QH, Qionghai; SY, Sanya; TC, Tunchang; WC, Wenchang; WN, Wanning; WZS, Wuzhishan.


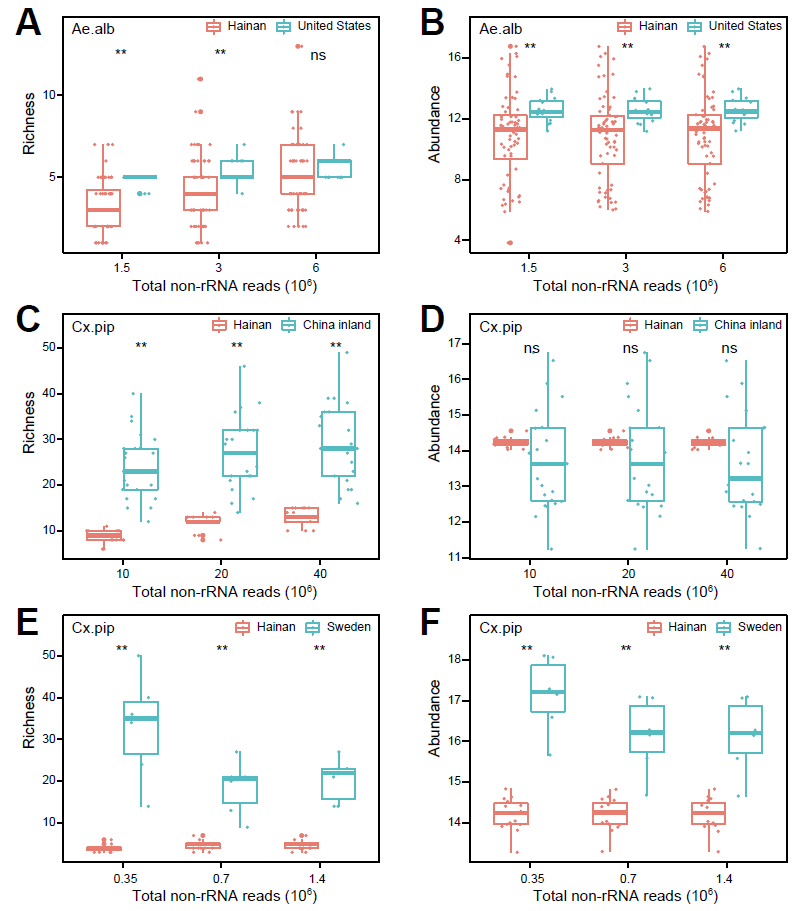


## Supplementary Figure 26. The observed richness of operational taxonomic units of RNA viruses (A, C, E) and RNA virus abundance (B, D, F) between Hainan Island and other collection locations in Yunnan (inland China, Asia), California (United States, North America) and Sweden (Europe). In pairwise comparisons, Wilcoxon rank sum tests were applied to detect significant differences. **P* < 0.05; ***P* < 0.01. Ae.alb, *Aedes albopictus*; Cx.pip, *Culex pipiens quinquefasciatus*.


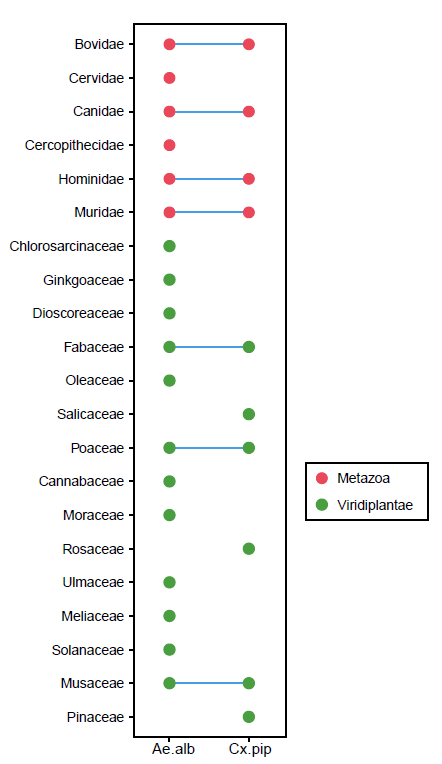


## Supplementary Figure 27. Bubble plot showing the distribution of food sources derived from animals and plants in *Aedes albopictus* and *Culex pipiens quinquefasciatus* in Hainan Island. The shared families are linked using blue lines. Ae.alb, *Aedes albopictus*; Cx.pip, *Culex pipiens quinquefasciatus*.


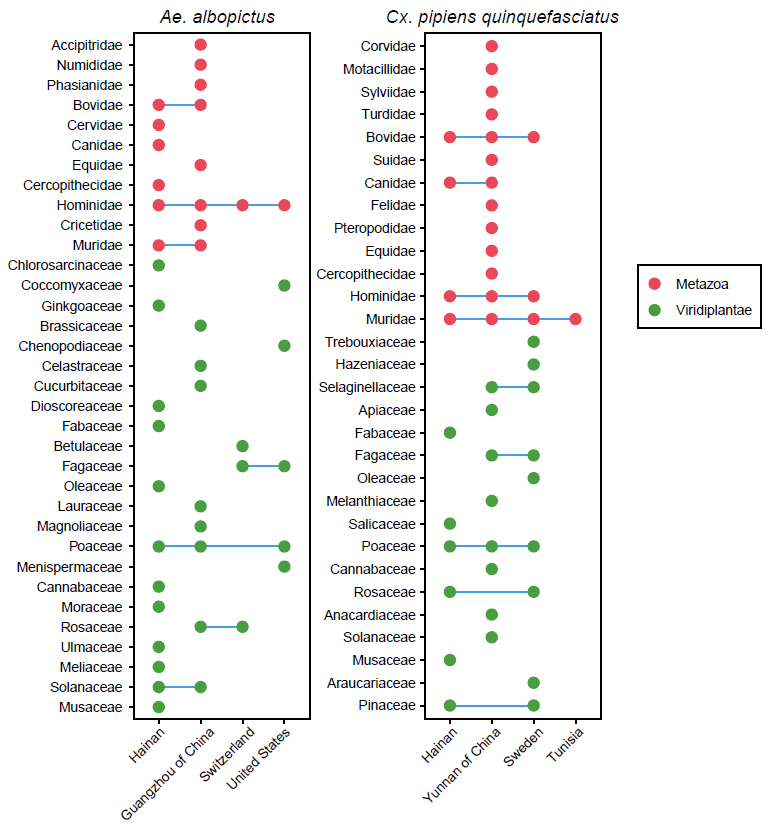


## Supplementary Figure 28. Bubble plot showing the distribution of food sources derived from animals and plants in *Aedes albopictus* (left) and *Culex pipiens quinquefasciatus* (right) in the four continental regions. The shared families are linked using blue lines.
